# Prenatal Arsenic Exposure Alters Gene Expression in the Adult Liver to a Proinflammatory State Contributing to Accelerated Atherosclerosis

**DOI:** 10.1371/journal.pone.0038713

**Published:** 2012-06-15

**Authors:** J. Christopher States, Amar V. Singh, Thomas B. Knudsen, Eric C. Rouchka, Ntube O. Ngalame, Gavin E. Arteel, Yulan Piao, Minoru S. H. Ko

**Affiliations:** 1 Department of Pharmacology and Toxicology, University of Louisville, Louisville, Kentucky, United States of America; 2 Center for Environmental Genomics and Integrative Biology, University of Louisville, Louisville, Kentucky, United States of America; 3 Center for Genetics and Molecular Medicine, University of Louisville, Louisville, Kentucky, United States of America; 4 Department of Molecular, Cellular and Craniofacial Biology, University of Louisville, Louisville, Kentucky, United States of America; 5 Department of Computer Engineering and Computer Science, University of Louisville, Louisville, Kentucky, United States of America; 6 Laboratory of Genetics, National Institute on Aging, Baltimore, Maryland, United States of America; The University of Arizona, United States of America

## Abstract

The mechanisms by which environmental toxicants alter developmental processes predisposing individuals to adult onset chronic disease are not well-understood. Transplacental arsenic exposure promotes atherogenesis in apolipoprotein E-knockout (ApoE^−/−^) mice. Because the liver plays a central role in atherosclerosis, diabetes and metabolic syndrome, we hypothesized that accelerated atherosclerosis may be linked to altered hepatic development. This hypothesis was tested in ApoE^−/−^ mice exposed to 49 ppm arsenic *in utero* from gestational day (GD) 8 to term. GD18 hepatic arsenic was 1.2 µg/g in dams and 350 ng/g in fetuses. The hepatic transcriptome was evaluated by microarray analysis to assess mRNA and microRNA abundance in control and exposed pups at postnatal day (PND) 1 and PND70. Arsenic exposure altered postnatal developmental trajectory of mRNA and microRNA profiles. We identified an arsenic exposure related 51-gene signature at PND1 and PND70 with several hubs of interaction (*Hspa8*, *IgM* and *Hnf4a)*. Gene ontology (GO) annotation analyses indicated that pathways for gluconeogenesis and glycolysis were suppressed in exposed pups at PND1, and pathways for protein export, ribosome, antigen processing and presentation, and complement and coagulation cascades were induced by PND70. Promoter analysis of differentially-expressed transcripts identified enriched transcription factor binding sites and clustering to common regulatory sites. SREBP1 binding sites were identified in about 16% of PND70 differentially-expressed genes. Western blot analysis confirmed changes in the liver at PND70 that included increases of heat shock protein 70 (Hspa8) and active SREBP1. Plasma AST and ALT levels were increased at PND70. These results suggest that transplacental arsenic exposure alters developmental programming in fetal liver, leading to an enduring stress and proinflammatory response postnatally that may contribute to early onset of atherosclerosis. Genes containing SREBP1 binding sites also suggest pathways for diabetes mellitus and rheumatoid arthritis, both diseases that contribute to increased cardiovascular disease in humans.

## Introduction

Environmental exposures during embryonic/fetal development have been shown to disturb the embryonic/fetal developmental program [1]. Some exposures cause severe disturbance of the program and result in gross structural anomalies. Others may have more subtle effects and may contribute to increased susceptibility to chronic adult diseases including cancer, diabetes and cardiovascular disease (CVD). The mechanism(s) underlying the effects of more modest environmental exposures on outcomes decades later are not understood.

The major cause of death in the U.S. is CVD. Atherosclerosis underlies most CVD which most often culminates in myocardial infarctions, strokes and renal disease. Inorganic arsenic (As) is a worldwide natural drinking water contaminant [2], [3] affecting over 140 million people in over 70 countries and is a high priority hazardous substance in the United States. Drinking water arsenic in third world countries is often above 100 µg/L and can be over 1 mg/L as in parts of West Bengal [4]. These high level exposures also occur in unregulated private well water in rural areas of the United States. In humans, arsenic exposure causes cancer, diabetes and atherosclerosis [2], [3], [5]–[7]. Arsenic can be teratogenic in rodents [8]. Epidemiological studies indicate that chronic arsenic ingestion causes CVD in humans (reviewed in [7]). A recent study showed that even very low arsenic exposure is associated with elevated CVD in Spain [9]. A West Bengali population with arsenic exposure had high rates of non-cirrhotic portal hypertension [10] which is consistent with recent findings of loss of hepatic sinusoidal fenestrae in arsenic exposed mice [11]. A recent longitudinal study found that the leading cause of death in arsenic exposed region of Bangladesh was CVD [12]. The incidence of CVD is increased in the U.S. when the drinking water As concentration exceeds 5 ppb [5], [6], [13], [14]. Thus, widespread exposure to arsenic in drinking water in the U.S. likely contributes to atherogenesis and death from CVD. Early life, particularly fetal, arsenic exposure contributes to development of CVD in adult life. Deaths from myocardial infarction caused by advanced arteriosclerosis of infants whose mothers consumed water with very high levels of arsenic clearly indicate that transplacental arsenic exposure is a risk factor for arterial disease in humans [15], [16]. Thus, it is clear that arsenic exposure contributes to CVD in adulthood, but the mechanism of arsenic-induced arterial disease is not known.

The interaction of *in utero* arsenic exposure with disease predisposition is not well understood. A transplacental arsenic exposure model of cancer induction in mice has been characterized and showed decreased latency and increased multiplicity of tumors in cancer prone C3H mice [17]. Whole life exposures (beginning preconception) induce tumors in CD1 mice which have a very low cancer background [18]. The studies to date in this model indicate that arsenic exposure alters gene expression in target tissues [19] and increases the stem cell population [20]. We developed an analogous model for *in utero* arsenic exposure induction of CVD. Our studies demonstrated that atherosclerosis in Apolipoprotein E-knockout (ApoE^−/−^) mice is both accelerated and exacerbated by transplacental arsenic exposure from gestational day 8 to birth [21]. *In utero* arsenic exposure reduced the time to detectable atherosclerotic disease to 10 weeks of age. Thus, the disease outcome of *in utero* arsenic exposure appears to be dependent upon the genetic predisposition to disease [22].

The liver plays a central role in the interlinked diseases diabetes mellitus, metabolic syndrome and atherosclerosis. Atherosclerotic changes in arsenic exposed ApoE-knockout mice are associated with decreases in plasma triglycerides [21]–[23]. The liver is the principal site of plasma triglyceride synthesis. Arsenic is a well known hepatotoxicant and causes fatty liver disease that can progress to cirrhosis. Arsenic exposure-induced liver disease is one example of environmental exposures leading to what has been termed toxicant associated steatohepatitis (TASH) [24]. Fatty liver disease is an independent risk factor for CVD (reviewed in [25], [26]). Thus, we reasoned that arsenic-induced liver disease is a likely cause of the accelerated atherogenesis we observe in ApoE^−/−^ mice. Furthermore, we hypothesized that transplacental arsenic exposure impacted liver development and that *in utero* arsenic exposure serves as the first hit in a two-hit model of liver injury that ultimately contributes to accelerated atherogenesis in arsenic exposed mice.

Hence, we sought to determine whether changes in gene expression could be detected that would correspond to altered development in livers of arsenic exposed mice. We show that changes in developmental trajectory are reflected in alterations in mRNA and microRNA expression patterns. These transplacental arsenic exposure induced liver gene expression changes also are linked to systemic inflammatory diseases associated with increased CVD in humans. Predictions from the transcriptomics data and their analyses are substantiated by biochemical and biological data suggesting long lasting stress response and subtle liver injury contributing to a pro-inflammatory state in the livers of exposed mice.

## Results

### Arsenic Exposure Model is Relevant to Human Populations with High Arsenic Exposure

**Figure 1:**
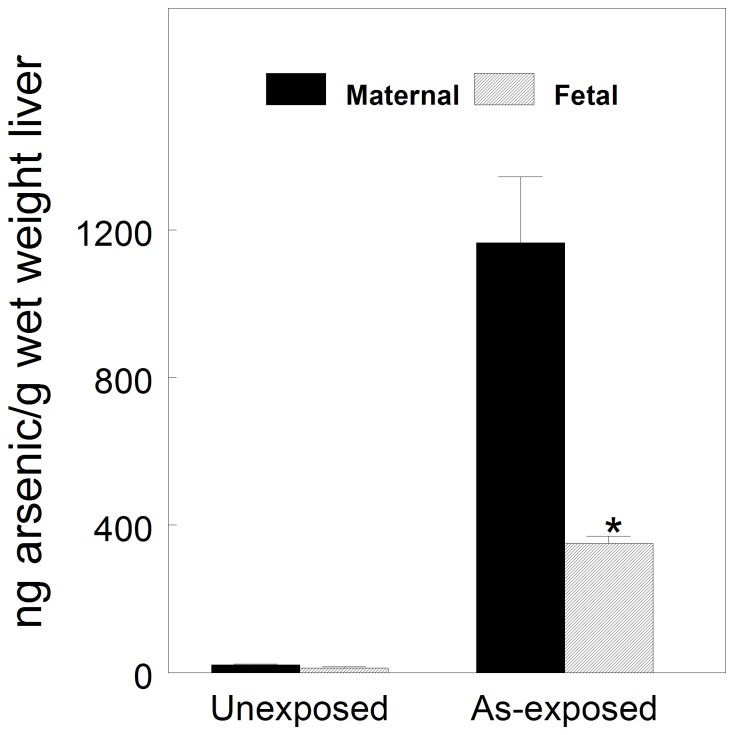
Determination of As levels in livers of pregnant dams and GD18 fetuses after 10 days exposure to 49 ppm As (as NaAsO2) in drinking water. Means±SD. P<0.01.

Phenotypic anchoring of the arsenic exposure model is important for relevance to human exposures. Drinking water arsenic exposures in West Bengal and other third world countries can be as high as 1 ppm (or even higher). The mean adult liver arsenic content in a sample of the arsenic exposed Bengali population was reported to be ∼1.5 mg/kg [10]. We measured arsenic levels in livers from exposed and unexposed pregnant dams and their fetuses at GD18. As shown in Figure 1, maternal arsenic exposure increased arsenic content in liver ∼55-fold to ∼1.2 µg/g wet weight, similar to that reported in the Bengali sample, and in the fetuses ∼26-fold to ∼350 ng/g. Thus, our animal model displays a key tissue phenotype that is consistent with human populations with high arsenic exposures. In addition, these results indicate that arsenic absorbed by the mother was crossing the placenta and getting to the fetal livers.

### mRNA and microRNA Expression Analyses

**Figure 2:**
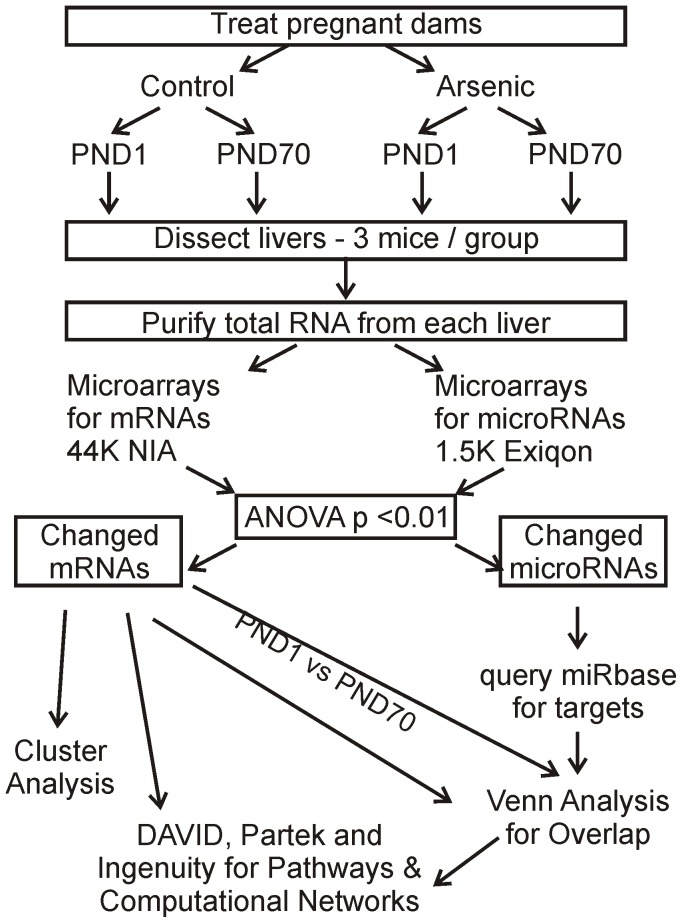
Diagram of experimental design and analytical flow for mRNA and miRNA data.

**Figure 3:**
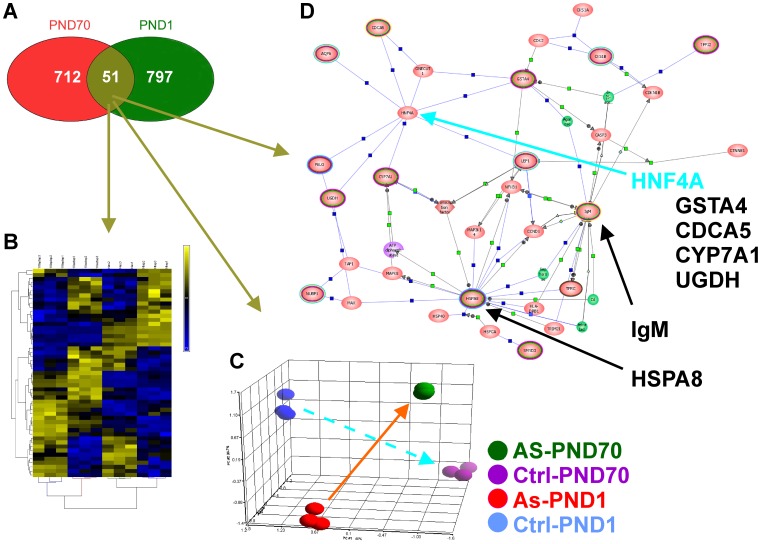
Identification and analyses of 51 genes differentially expressed at both PND1 and PND70. A. Intersection of genes differentially expressed at PND1 and PND70. B. Hierarchical clustering of 51 genes differentially expressed at both PND 1 and PND70. C. Principal component analysis of 51 genes differentially expressed at both PND 1 and PND70. (Arrows added for clarity) D. Pathway Architect analysis of 51 genes differentially expressed at both PND 1 and PND70.

The expression of most genes in a pathway changes little when a pathway is induced because only a few are actually rate limiting. However, in order to identify an affected pathway in biological function analyses, one must have a sufficient number of genes differentially expressed to detect an enriched pathway. In addition, we were analyzing total liver RNA and liver is composed of multiple cell types, each making their contribution to the total RNA pool. Our plan included confirmation of conclusions from genomics analyses with biochemical or biological assays post-hoc. Hence, we sought to identify all genes with changed expression and set the criteria accordingly. Total RNAs were prepared from liver samples from pups prenatally exposed to arsenic and evaluated on day of birth (PND1) and 10-weeks of age (PND70) (n = 3 for each group). The experimental design and workflow for the RNA analyses is shown in Figure 2. RNAs were prepared and analyzed by microarrays as described in methods. We identified a total 848 probes mapping to 797 individual genes with expression changed by in utero arsenic exposure at PND1 and 763 probes mapping to 712 individual genes at PND70 (p<0.01, any change; Figure 3A).

### Arsenic Exposure Differential Gene Expression Signature

We next sought to identify a postnatal signature modified by prenatal arsenic exposure. Comparative analysis of differentially-expressed mRNAs at PND1 and PND70 revealed a 51 gene signature with significantly altered expression at both life stages (Figure 3A). Unsupervised cluster analysis further classified these genes into four major groups. Some genes had increased expression at both time points, some were decreased at both time points, some were up at one and down at the other time point (Figure 3B).

Principal component analysis of the 51-gene signature set showed the major explained variance was due to life stage. This was followed by treatment (Figure 3C). However, the postnatal developmental trajectories were markedly different in arsenic exposed and unexposed animals. This implies a shift in fetal programming due directly or indirectly to the maternal arsenic intake.

Analysis with Pathway Architect (Figure 3D) identified three major hubs in the interactome of the 51 gene signature (Table 1): HspA8, IgM and HNF4A. Hspa8 is heat shock 70 kDa protein 8, a cognate heat shock protein, and its mRNA was induced at both PND1 and PND70. In contrast, IgM, the immunoglobulin light chain variable region, is suppressed at both PND1 and PND70. HNF4A is a hub, but its mRNA was unchanged. However, HNF4A is known to regulate LEF1, GSTA4, CDCA5, CYP7A1 and UGDH which were induced or suppressed by prenatal arsenic exposure (Table 1).

**Table 1:** Network analysis of 51 gene signature.

Gene	Description	Change in expression	
		PND1 As	PND70 As
Hspa8	Cognate HSP70	Up	Up
Igm	Immunoglobulin light chain variable region	Down	Down
Hnfa	controls liver expression of several genes including the following:	NC	NC
Lef1	lymphoid enhancer-binding factor 1	Up	Down
Aqp1	water channel	Up	Down
Gsta4	glutathione S-transferase A4	Down	Up
Cdca5	regulator of sister chromatid cohesion	Down	Up
Cyp7a1	rate limiting step and the major site of regulation of bile acid synthesis	Down	Up
Ugdh	biosynthesis of glycosaminoglycans	Down	Up

NC = not changed.

### Differential Gene Expression Analyses Suggest Activation of Inflammatory Pathways

**Figure 4:**
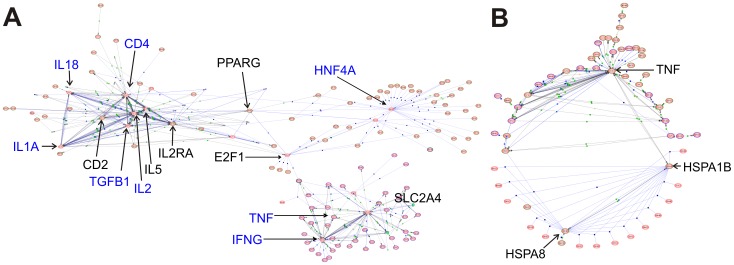
Interactome maps of differentially expressed mRNAs in livers of arsenic-exposed mice. A. PND1; B. PND70. Major hubs are annotated.

Interactome analyses of the entire sets of differentially expressed mRNAs at PND1 (Figure 4A) and PND70 (Figure 4B) revealed the HNF4A hub, along with elevation of a variety of inflammatory cytokines. HNF4A also is a major hub in the interactome of mRNAs suppressed at PND70 (Figure S1). The network linkages to HNF4A in the PND70 interactome are genes not traditionally associated with inflammation. HspA8 (HSC70) and HspA1B (HSP70) were major hubs in the PND70 interactome. Thus, the 51-gene signature identified major hubs present in the individual interactomes at each age. Tnf, a multifunctional proinflammatory cytokine secreted mainly by macrophages, was a major hub in both the PND1 and PND70 total interactomes (Figure 4). Tnf was unchanged in PND1 but induced in PND70 livers, and was a major hub in the interactome of genes induced in livers of arsenic exposed PND70 mice with links to several induced inflammatory cytokines (Figure S2). These results are consistent with a pro-inflammatory state at PND70.

MicroRNAs (miRNAs) are believed to play a role in developmental buffering and pathway regulation. We thus profiled the miRNA transcriptome to determine if developmental alterations would be reflected in the liver at PND1 and PND70. We identified 31 annotated microRNAs differentially expressed at either time point by arsenic exposure. The mRNA targets of these microRNAs were queried from the Sanger miRbase database. Three miRNAs were induced and eight were suppressed in livers of arsenic exposed PND1 mice (Table 2). Scanning the miRBase with three induced miRNAs resulted in 2413 non-redundant targets (2813 redundant targets) whereas scanning with the eight suppressed microRNAs resulted in 4994 non-redundant targets (6780 non-redundant). There were 1038 common probes between targets of three induced and eight suppressed PND1 microRNAs. Five microRNAs were induced and 15 were suppressed (Table 2) in livers of arsenic exposed PND70 mice. Scanning miRBase for target mRNAs with the five induced microRNAs resulted in 2669 non-redundant targets (3579 redundant targets) whereas 15 suppressed microRNAs had 8246 non-redundant targets (13142 non-redundant). There were 1856 common probes between targets of five induced and 15 suppressed PND70 microRNAs. There was no uniform correlation in differential expression between the targets for these microRNAs and differentially expressed mRNAs. We did see both induction and suppression of the target mRNA transcripts.

**Table 2:** microRNAs differentially expressed at PND1 and PND70.

microRNAs inducedat PND1	microRNAs inducedat PND70
mmu-miR-361	mmu-miR-211
mmu-miR-148a	mmu-miR-291a-5p-291b-5p
mmu-miR-467a	mmu-miR-294
	mmu-miR-302
	mmu-miR-464
**microRNAs suppressed** **at PND1**	**microRNAs suppressed** **at PND70**
mmu-miR-188	mmu-miR-1
mmu-miR-211	mmu-miR-10a
mmu-miR-222	mmu-miR-15b
mmu-miR-497	mmu-miR-124a
mmu-miR-592	mmu-miR-130a
mmu-miR-679	mmu-miR-149
mmu-miR-719	mmu-miR-184
mmu-let-7d	mmu-miR-193
	mmu-miR-218
	mmu-miR-337
	mmu-miR-376a
	mmu-miR-412
	mmu-miR-467b
	mmu-miR-681
	mmu-miR-715

Developmental miRNA expression does not necessarily have an inverse correlation with target gene expression. Both coherent and non-coherent expression is found in developmental systems [27], [28]. Hence it was not surprising that we did not find an inverse correlation between mRNAs identified by the intersection of the differentially expressed mRNAs and the target mRNAs of the differentially expressed microRNAs. GO annotation analysis of the differentially-expressed miRNA targeted mRNAs were analyzed by DAVID to identify which pathways were represented among the set. The results indicate that fundamental metabolic processes (glycolysis/gluconeogenesis) were decreased in livers of arsenic-exposed PND1 mice, and that protein metabolism (protein export, ribosome) and inflammatory processes (complement, antigen processing, coagulation) were increased in livers of arsenic-exposed PND70 mice (Table 3). These pathways also are represented in the GO analyses of total mRNAs induced or suppressed at PND1 or PND70 (Tables S1, S2, S3, S4).

**Table 3:** Gene ontology of differentially expressed microRNA target mRNAs.

Down by Arsenic PND1 (p<0.05)			
Category	Term	Count	Pvalue
KEGG_PATHWAY	MMU00010:Glycolysis/Gluconeogenesis	6	0.005
**Up by Arsenic PND70 (p<­0.05)**			
**Category**	**Term**	**Count**	**Pvalue**
KEGG_PATHWAY	MMU03060:Protein Export	4	6.76E-04
KEGG_PATHWAY	MMU03010:Ribosome	6	0.022
BIOCARTA	M_lectinPathway:Lectin Induced Complement Pathway	3	0.016
KEGG_PATHWAY	MMU04612:Antigen Processing and Presentation	6	0.019
BIOCARTA	M_classicalPathway:Classical Complement Pathway	3	0.028
KEGG_PATHWAY	MMU04610:Complement and Coagulation Cascades	5	0.033
BIOCARTA	m_compPathway:Complement Pathway	3	0.034

### Identification of Common Transcription Factors Potentially Regulating the Changes in Expression Profiles

We searched for potentially coordinately regulated sets of genes by performing transcription factor binding site (TFBS) analysis of the differentially expressed genes. The sequences of the regions 2 kb 5′ plus 200 bp 3′ to the transcription initiation site of each gene were collected from Genbank. These sequences were submitted to the Transfac database [29] to identify TFBS and then analyzed using Expander to identify TFBS enrichment (Tables S5, S6, S7, S8, S9, S10, S11, S12). A total of 760 unique Entrez gene IDs were differentially expressed mRNAs at PND1 and a total 18 TFBS were enriched in this gene set (P<0.05). Likewise, a total of 690 unique Entrez gene IDs were differentially expressed mRNAs at PND70, and a total 31 TFBS were enriched in this gene set (P<0.05). Among the enriched TFBS identified are several that are particularly pertinent to this discussion. These include the developmental regulatory factor homeobox C8 (HOX3A), stress response factors heat shock factor (HSF), nuclear factor of kappa light polypeptide gene enhancer in B-cells 1 (NFKB) and activator protein 1 (AP1), environmental agent response factors arylhydocarbon receptor (AHR) and hypoxia inducible factor (HIF1), cell cycle regulatory factor E2F transcription factor 1 (E2F1), the lipid metabolism regulator gene sterol regulatory element binding protein 1 (SREBP1), and chromatin remodeling factor p300 among others (Tables 4 and 5, and S5, S6, S7, S8, S9).

**Table 4:** Transcription factor binding sites enriched in gene promoters of differentially expressed mRNAs in PND70 mice.

TranscriptionFactor	Numberof Genes	P-Value	EnrichmentFactor
M00025[Elk-1]	97	2.46E-05	1.337
M00024[E2F]	42	6.46E-04	1.644
M00056[myogenin_/_NF-1]	56	0.001	1.371
M00341[GABP]	109	0.001	1.257
M00678[Tel-2]	59	0.002	1.389
M00652[Nrf-1]	130	0.003	1.191
M00801[CREB]	67	0.004	1.221
M00492[STAT1]	56	0.004	1.409
M00938[E2F-1]	134	0.004	1.223
M00803[E2F]	197	0.004	1.202
M00940[E2F-1]	62	0.005	1.401
M00449[Zic2]	56	0.007	1.341
M00807[EGR]	149	0.008	1.159
M00196[Sp1]	254	0.009	1.133
M00017[ATF]	96	0.01	1.276
M00531[NERF1a]	83	0.014	1.276
M00647[LXR]	77	0.014	1.319
M00373[Pax-4]	102	0.02	1.234
M01045[AP-2alphaA]	64	0.024	1.146
M00243[Egr-1]	121	0.024	1.189
M00430[E2F-1]	46	0.027	1.249
M00235[AhR:Arnt]	65	0.028	1.226
M00915[AP-2]	146	0.029	1.163
M00749[SREBP-1]	113	0.03	1.155
M00224[STAT1]	49	0.032	1.398
M01033[HNF4]	233	0.032	1.086
M00055[N-Myc]	87	0.034	1.249
M00237[AhR:Arnt]	76	0.036	1.196
M00033[p300]	53	0.038	1.26
M00695[ETF]	132	0.041	1.151
M00778[AhR]	82	0.049	1.219

A total of 690 unique entrez gene IDs are differentially expressed mRNAs at PND70. A total 31 transcription factors are enriched for this gene set.with a P-value <0.05.

**Table 5:** Transcription factor binding sites enriched in gene promoters of differentially expressed mRNAs that are targets of differentially expressed microRNAs in PND1 mice.

TranscriptionFactor	Numberof Genes	P-Value	EnrichmentFactor
M00649[MAZ]	28	0.003	1.72
M00490[Bach2]	17	0.004	2.2
M00731[Osf2]	13	0.006	2.304
M00467[Roaz]	12	0.007	1.779
M00976[AHRHIF]	27	0.009	1.556
M00172[AP-1]	15	0.009	1.826
M00395[HOXA3]	8	0.009	1.982
M00076[GATA-2]	13	0.021	2.012
M01033[HNF4]	47	0.022	1.176
M00678[Tel-2]	12	0.027	1.517
M00797[HIF-1]	28	0.031	1.547
M01109[SZF1-1]	14	0.031	1.59
M00652[Nrf-1]	28	0.036	1.377
M00205[GR]	11	0.04	1.743
M01045[AP-2alphaA]	18	0.042	1.731
M00484[Ncx]	12	0.042	1.859
M00378[Pax-4]	21	0.046	1.343

A total of 101 unique Entrez gene IDs are gene targets of induced or suppressed microRNAs and appear in the gene list of differentially expressed mRNAs at PND1. A total 17 transcription factors are enriched for this gene set with a P-value <0.05.

**Figure 5:**
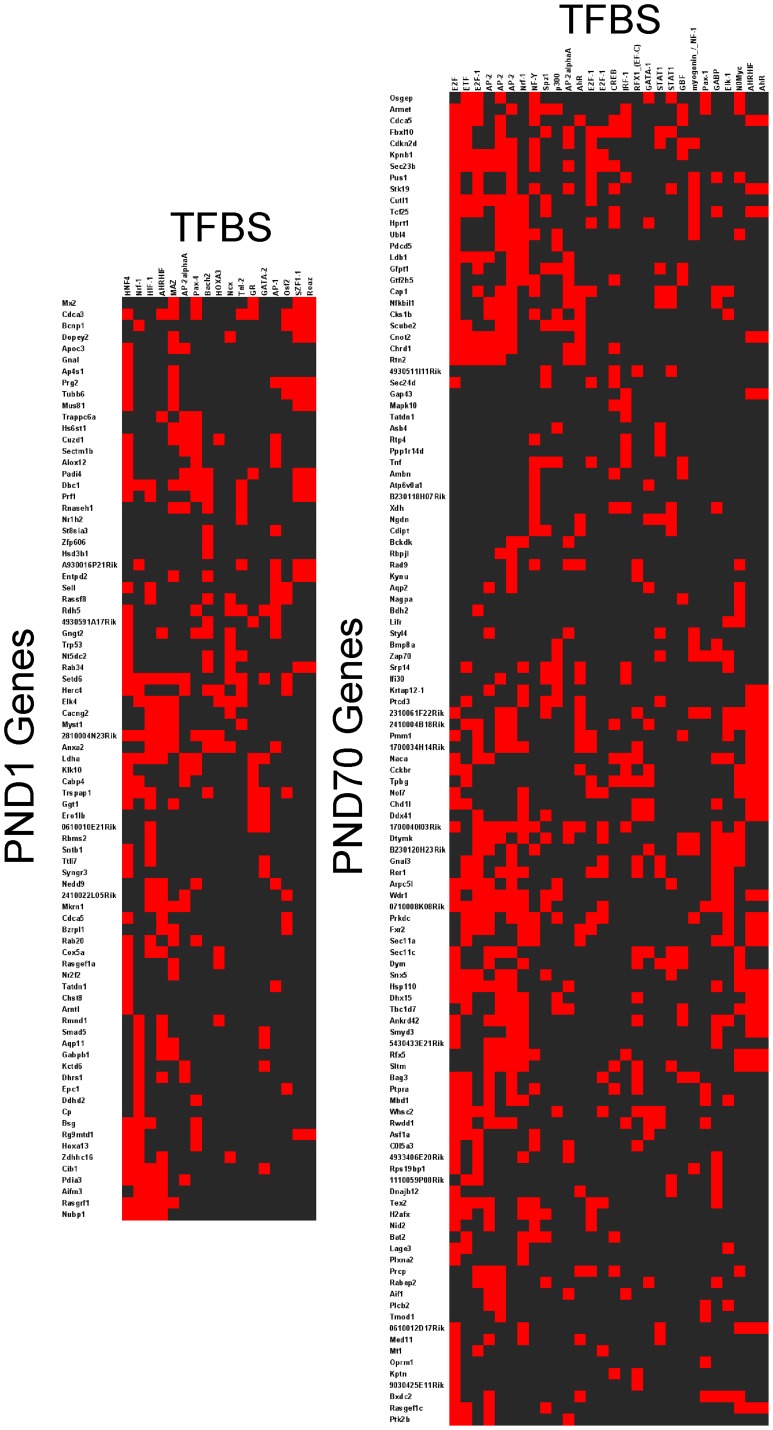
Cluster maps of enriched transcription factor binding sites (TFBS) in promoters of genes that are differentially expressed at PND1 (101 genes) and PND70 (135 genes) and also are targets of differentially expressed microRNAs at PND1 (Table S6) and PND70 (Table S11) respectively.

Table 5 lists the differentially-expressed genes at each life stage that also were targets of differentially-expressed miRNAs at that age and that contained the enriched TFBS present in their promoters. Assuming genes with shared multiple TFBS are coordinately regulated, we noted (Figure 5) the upper left of the PND70 map showing 7 genes (*Osgep, Armet, Cdca5, Cdkn2d, Kpnb1, Sec23b*) sharing 4 TFBS (E2F1, ETF, Ap2, NFY). In addition, 113 genes with altered expression at PND70 had binding site(s) for SREBP1 (Table 4). SREBP1 target genes were not, however, represented among the differentially-expressed miRNAs. The large fraction of the genes with altered expression, all with a common TFBS, thus suggests that SREBP1 is activated and perhaps coordinately regulates this network of genes.

### In Utero Arsenic Exposure Induces Changes in Protein Expression in Adult Livers that Confirm Inferences from the mRNA Abundance and TFBS Analyses

**Figure 6:**
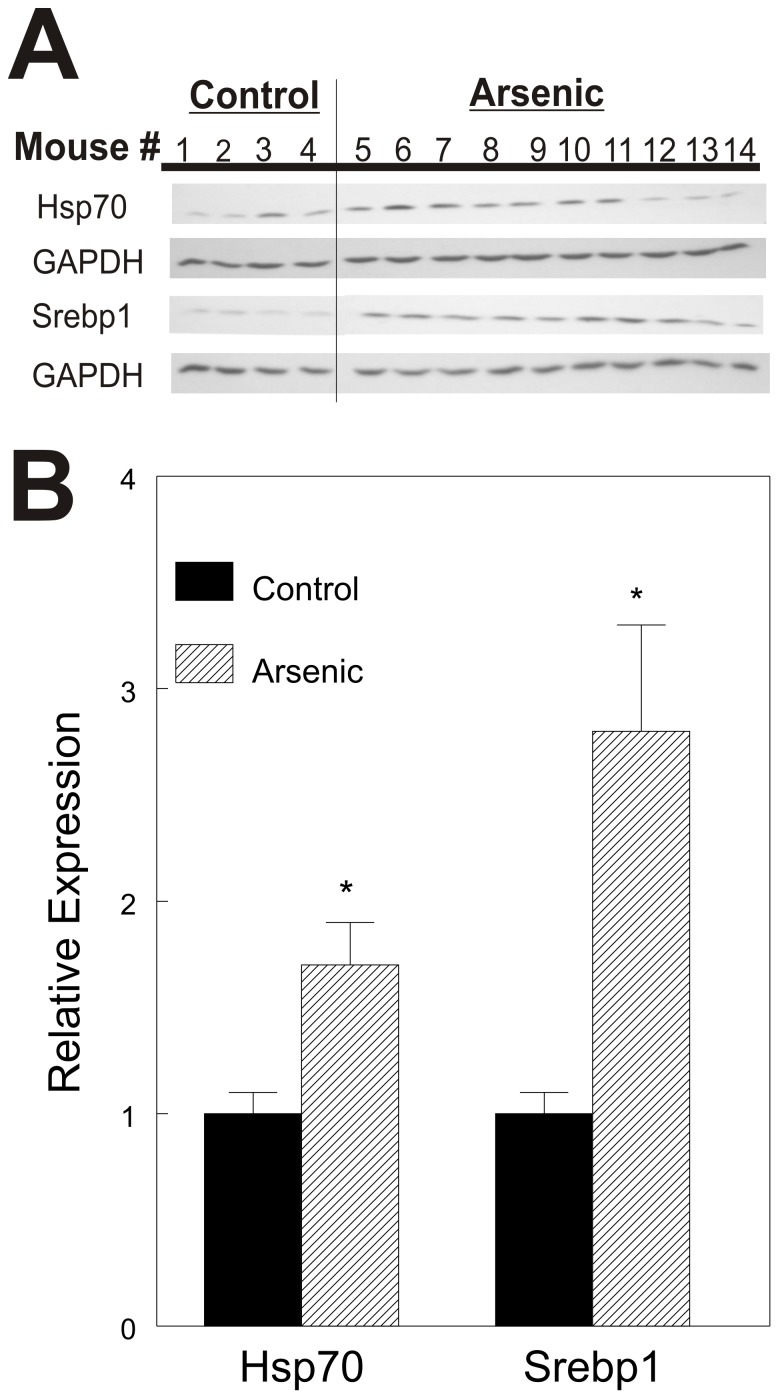
HSP70 and SREBP1 expression in livers of 10 week old mice. A. Western blot analysis of HSP70 and SREBP1. The SREBP1 fragment shown is the 68 kDa cleavage fragment. B. Densitometric quantitation of western blots. Signals for each protein were normalized to GAPDH and mean of controls (#’s 1–4) was set = 1. Representative of triplicate blots shown. * = p<0.05.

The mRNA expression data indicated that the genes for heat shock 70 proteins, HSP70 (HSPA1B, inducible form) and HSC70 (HSPA8, constitutive form), were induced. TFBS analyses indicated that SREBP1 binding sites were enriched in the promoters of differentially expressed genes in PND70 mice suggesting potential coordinate induction by SREBP1. However, SREBP1 mRNA level was unchanged. Because, SREBP1 activity is dependent upon cleavage in the endoplasmic reticulum, we examined expression of HSP70, HSC70 and the active form of SREBP1 by western blot analysis. The data (Figure 6) indicate that levels of HSP70 and activated SREBP1 are elevated in livers of As-exposed mice. Thus, the prediction made from the TFBS analysis, that activated SREBP1 is likely elevated because a battery of differentially expressed genes have an SREBP1 binding site in their promoters, is confirmed at the protein level. HSC70 did not change with arsenic exposure (not shown). The induction of HSP70 and activated SREBP1 in PND70 mice suggests a mild stress following prenatal arsenic exposure enduring into adult life. These results support the inferences made from the bioinformatics analyses of the mRNA microarray data.

### Disease Functions Identified in Srepb1 TFBS Containing Gene Set

**Figure 7:**
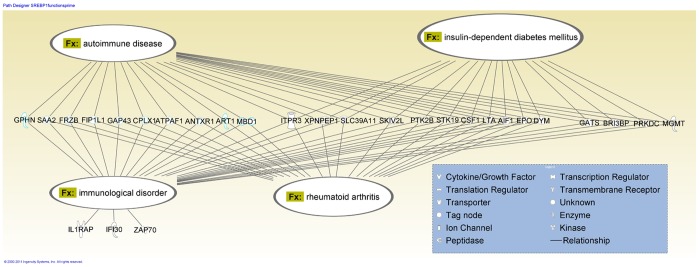
Analysis of SREBP1 gene network for disease functions.

The induction of activated SREBP1 in the face of unchanged SREBP1 mRNA and reduced plasma triglycerides implies a dysregulation of lipid metabolism and/or transport. The 113 genes with SREBP1 TFBS were analyzed for interaction networks and disease functions using IPA software. The results revealed that this set of genes contained subnetworks of genes associated with immunological disorder, autoimmune disease, rheumatoid arthritis and insulin dependent diabetes mellitus (Figure 7). These subsets of genes overlap with one another consistent with the obvious relatedness of the first three subsets. Induction of these gene sets also is consistent with a pro-inflammatory state.

### Subtle Liver Injury Persists Long After Arsenic Exposure

**Figure 8:**
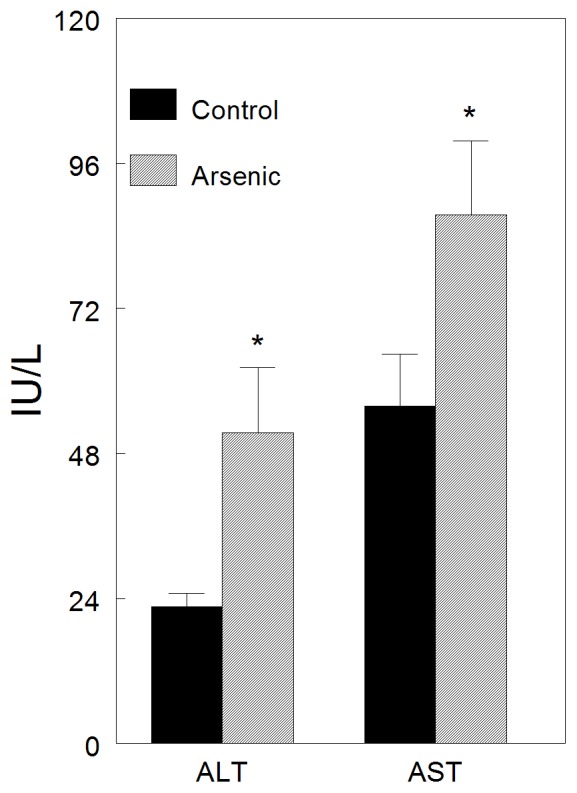
Plasma ALT and AST in arsenic-exposed and -unexposed PND70 ApoE-knockout mice. Mean±S.E. n = 8−9 (*p<0.026; #p = 0.056).

The microarray data suggested that inflammatory cytokines were elevated in livers of newborn (PND1) mice and that the stress response was activated in PND70 livers. In particular, tumor necrosis factor alpha (TNFΑ) expression was elevated in PND70 mice and TNFΑ has been linked to liver inflammation [30]. Therefore, we examined the livers of arsenic exposed and unexposed mice at 10 weeks of age to determine whether signs of inflammation were present. Histological assessment of liver tissues stained with hematoxylin and eosin revealed no macroscopic histological changes in the livers of arsenic-exposed mice (not shown). However, liver injury may be subtle. Therefore, we determined levels of circulating liver enzymes as clinical markers of liver injury. Plasma levels of both ALT and AST determined using commercially available kits were elevated in arsenic-exposed mice suggesting liver damage (Figure 8). These results support the gene expression data suggesting elevated liver inflammation and clearly indicate that prenatal arsenic exposure causes subtle (but significant) liver damage that may prime the liver for an inflammatory stimulus.

## Discussion

In the current study, we used a transcriptomics approach to investigate dysregulation of liver development induced by prenatal arsenic exposure in ApoE-knockout mice. Differential gene expression at birth (immediately after the exposure) and at age 10 weeks (when accelerated atherogenesis is first detectable [21]) showed that the liver developmental program was dysregulated. A 51-gene expression signature of arsenic-induced developmental changes was identified. Increased expression of inflammatory cytokine and heat shock protein 70 (HSP70) mRNAs in livers of 10 week old mice suggested an enduring state of stress and the presence of subtle liver injury. Promoter analyses for TFBS in differentially expressed genes suggested increased expression of developmental and stress related transcription factors. Key inferences from the transcriptomic analyses (e.g. stress, transcription factor activation, liver injury) were supported by biochemical and biological assays showing elevated hepatic HSP70 and mature SREBP1 expression, and elevated liver enzymes, ALT and AST, in plasma.

Prenatal arsenic exposure induced differential gene expression in newborn (PND1) and 10 week old (PND70) mice. The principal component analysis indicated that the gene expression profiles of PND1 and PND70 livers were altered by arsenic exposure and age indicating that the normal age-dependent changes in expression profiles were dysregulated by the in utero arsenic exposure. This dysregulation of developmental program caused long lasting changes in livers of exposed mice as indicated by identification of HNF4A, TNF, HSPA8 (HSC70) and HSPA1B (HSP70) identified as major hubs of interaction in differentially expressed genes at both PND1 and PND70. The gene expression changes associated with major hubs in the interactome analyses are consistent with the accelerated atherogenic phenotype of arsenic exposed mice. HNF4A (hepatocyte nuclear factor alpha) is a major hub in the 51 gene arsenic signature as well as in both the PND1 and PND70 interactomes. HNF4A transcription factor binding sites were enriched in the promoters of genes with arsenic-induced differential expression at PND70 (Table 4) and PND1 differentially expressed gene targets of down regulated microRNA (Table 5). In humans, HNF4A gene variants are associated with type 2 diabetes mellitus [31]. HNF4A plays a role in regulating the folate receptor locus [32]. Folate receptor 2 (FOLR2) mRNA was induced in arsenic-exposed PND70 mice (not shown). FOLR2 deficiency aggravates developmental consequences of arsenic exposure [33], so FOLR2 induction may be a compensatory response to arsenic exposure.

HNF4A mRNA was not changed in the microarray analyses but several of the genes it regulates exhibited altered expression (Table 1). These genes (lymphoid enhancer-binding factor 1 [*Lef1*], glutathione S-transferase alpha 4 [*Gsta4*], cell division cycle associated 5 [*Cdca5*], cytochrome P450 7A1 [*Cyp7a1*], pelota homolog [*Pelo*], aquaporin 6 [*Aqp6*] and UDP-glucose 6-dehydrogenase [*Ugdh*]) are involved in T-cell receptor regulation, xenobiotic detoxification, cell cycle, cholesterol metabolism and extracellular matrix biosynthesis. GSTA4, CYP1A7 and UGDH mRNAs were decreased in PND1 livers and increased in PND70 livers. GSTA4 and CYP1A7 are xenobiotic metabolizing enzymes. GSTA4 is involved in detoxification of lipid peroxidation products and has been linked to atherosclerosis [34]. CYP1A7 is the rate limiting enzyme in bile acid synthesis from cholesterol. UGDH converts UDP-glucose to UDP-glucuronate playing a role in the biosynthesis of glycosaminoglycans. AQP6 and LEF1 expression changes were the reverse of GSTA4, CYP1A7 and UGDH. AQP6 and LEF1 mRNAs were increased in PND1 livers and decreased in PND70 livers. AQP6 expression is reported to be kidney specific [35]. Thus, elevated AQP6 mRNA expression in newborn livers is consistent with developmental dysregulation. Lef1 is a lymphoid enhancer binding factor expressed in pre-B and pre-T cells [36]. Its elevated expression in newborn livers suggests activation of lymphoid cells and could contribute to an inflammatory response. Nucleotide binding protein 1 [NUBP1] showed expression patterns similar to AQP6 and Lef1 but is only indirectly related to the HNF4A network. NUBP1 has been implicated in the regulation of centrosome duplication [37] and is indirectly linked to HSPA8 in the network. We have shown that arsenite causes centrosome amplification similar to that induced by heat shock [38]. PELO also is in the HNF4A transcriptional network. Similar to HSPA8, PELO mRNA is increased in both PND1 and PND70 livers. PELO is an essential gene that has been implicated in controlling aneuploidy [39]. Arsenic is a well established aneuploidogen [40]. Thus, induction of both NUBP1 and PELO may be a compensatory response to the aneuploidogenic stimulus of arsenic. HNF4A regulates a common network of genes in liver and pancreas that when dysregulated may contribute to development of diabetes mellitus [41]. The incidence of diabetes mellitus in arsenic exposed populations is elevated [42], [43]. Hence, our results suggest that the mode of action for arsenic exposure inducing diabetes mellitus may involve its effects on the HNF4A network in addition to the potential role of the SREBP1 network (discussed below).

HSPA8 (HSC70) and HSPA1B (HSP70) were major hubs in the PND1 interactome of differentially expressed mRNAs that are targets of differentially expressed microRNAs. These genes encode the constitutive and inducible isoforms of heat shock protein 70 (HSC70 and HSP70 respectively). HSC70 has been associated with stabilization of newly synthesized cyclin D1 and is a component of active cyclin D1/CDK4 holoenzyme complex [44]. Prenatal arsenic exposure induces cyclin D1 in livers of several strains of mice [18], [45]. HSP70 induction is a well recognized response to cellular stress induced by a wide variety of agents. Circulating HSP70 induces a pro-inflammatory state [46], activates macrophages [47], can serve as a cytokine for both innate and adaptive immunity [48], and is associated with increased risk of acute coronary syndrome [49]. Our data indicate induced expression of HSP70 in livers of arsenic-exposed mice (Figure 6), thus confirming the inference from the transcriptomics data. If exported into the circulation, this enhanced HSP70 expression likely contributes to the accelerated atherogenesis observed in arsenic exposed ApoE-knockout mice.

TNF (tumor necrosis factor alpha, TNFΑ) is a major hub in both PND1 and PND70 differentially expressed mRNAs that are targets of differentially expressed microRNAs. TNF is an immunomodulator and stimulates the acute phase response. TNF expression was decreased at PND1 and increased at PND70 in arsenic exposed mice. TNF is linked to LTA (*aka* TNFB) and LTB (*aka* TNFC) in the PND70 interactome of differentially expressed mRNAs that are targets of differentially expressed microRNAs. LTA and LTB are members of the tumor necrosis factor superfamily expressed by B and T lymphocytes. TNF, LTA and LTB are involved in regulating a wide variety of immune responses and inflammation [50], [51]. The increased expression of this triad of inflammation mediators at PND70 is consistent with an inflammatory response occurring in livers of arsenic exposed PND70 mice.

Kupffer cells are the resident macrophages in the liver, are the principal players in the innate immune system, and are a major source of circulating cytokines including Interleukin 6 (IL6), Interleukin 10 (IL10), TNFA and monocyte chemoattractant protein-1 (MCP-1) [52]. Kupffer cells release MCP-1 after trauma and this MCP-1 plays a major role in remote organ dysfunction after trauma-hemorrhage [53]. Hepatic stellate cells are also toll-like receptor 4 (TLR4) positive cells and secrete the inflammatory cytokines IL6, IL8, MCP-1 and RANTES in response to LPS [54]. Both IL6 and IL8 are linked to TNF, and also to NFKB, in the PND70 interactome, as are LTA and LTB. IL8 attracts neutrophils which can contribute to the liver damage; RANTES attracts memory T-cells. Lipopolysaccharide response is mediated by activated TLR4 which is present in both Kupffer cells and hepatic stellate cells. TLR4 also is linked to TNF, IL6, IL8 and NFKB in the interactome (Figure S2). Thus, both these cell populations may contribute to circulating levels of cytokines that contribute to activation of monocytes in the initiation of atherogenesis. We have shown that chronic arsenic exposure for 12 weeks in adult mice elevates plasma MCP-1 and IL6 [55], both cytokines that are associated with atherosclerosis.

Analysis of TFBS in promoters of genes differentially expressed in 10 week old mice exposed to arsenic *in utero* revealed a number of enriched sites suggesting networks of genes coordinately regulated by the associated transcription factors. Stress response factors HSF1, NFKB and AP1 were among the transcription factors with binding sites enriched in the differentially expressed PND1 mRNAs. AP1 and NFKB were linked in a subset of 12 genes that IPA associated with inflammatory diseases including rheumatic disease and diabetes mellitus (not shown). Fry et al observed a network of genes with differential expression integrating NFKB in cord blood leukocytes of infants exposed to high levels of arsenic *in utero* [56]. This network included a sub-network centered around the pro-inflammatory interleukin I family member IL1B. We saw that IL18, an inflammatory cytokine structurally similar to IL1B, was a major hub in the interactome in our arsenic exposed PND1 mice (Figure 4A). Arsenite exposure induces HSP70 expression via activation of HSF1 [57]. Our microarray data indicate that HSP70 mRNA is induced and that a network of genes with HSF1 binding sites in their promoters is differentially expressed at both PND1 and PND70. Thus, the inferences made from the TFBS analyses are consistent with observations made by others both in humans and *in vitro*.

The TFBS analysis also showed that approximately 1/6 of the genes differentially expressed at PND70 had SREBP1 binding sites. This observation suggested that SREBP1 might be activated in the livers of arsenic exposed mice and we confirmed elevated levels of activated SREBP1 by western blot analyses. Thus, the power of the genomics approach to elucidate potential mechanisms is clearly exemplified by the biochemical confirmation of activated SREBP1 induction. Further study of the other inferred activated transcription factors is needed to gain a more complete understanding of the long lasting effect of the prenatal arsenic exposure on gene expression and liver development.

SREBP1 has important functions in regulating cholesterol and triglyceride synthesis, adipogenesis and insulin action [58]. Insulin induces SREBP1 in liver and adipocytes [59]–[61]. SREBP1 is responsive to insulin and glucose stimulation and regulates a plethora of genes in human HepG2 cells [62]. SREBP1 expression is reduced in human adipocytes and skeletal muscle in obese and insulin resistant people but can be returned to normal by hyperinsulinemia [63]. Hence, SREBP1 is intimately involved in lipid metabolism and is dysregulated in diabetic and pre-diabetic patients, both conditions linked to elevated CVD incidence. Our analysis revealed that SREBP1 likely also regulates a battery of genes not involved with lipid biosynthesis and not previously known to be SREBP1 regulated genes. Analysis of this set of genes revealed linkages to inter-related disease processes rheumatoid arthritis and insulin dependent diabetes mellitus. Both of these inflammatory diseases are associated with accelerated atherosclerosis [64]. Rheumatoid arthritis is the paradigm of a chronic systemic inflammatory disease. Rheumatoid arthritis patients exhibit greatly increased incidence of CVD [65]. Metabolic syndrome, a consequence of complex interactions between liver and adipose tissue, is well established as a risk factor for atherosclerosis leading to cardiovascular diseases. Metabolic syndrome has been proposed to provide linkage between rheumatoid arthritis and CVD [66]. The observed relationship of diabetes mellitus genes and rheumatoid arthritis genes supports an earlier suggestion that these disorders are linked [67]. Increased risk of CVD is a well established consequence of diabetes mellitus [68]. Our results suggest a role for activated SREBP1 in the etiology of these chronic systemic inflammatory diseases. This systemic inflammation likely contributes to the accelerated atherogenic phenotype of the prenatal arsenic exposed ApoE-knockout mice. Thus, the complex nature of the interactions between systemic inflammation and atherogenesis are reflected in our prenatal arsenic exposure model. It is important to confirm SREBP1 occupancy of the promoters in these genes in future studies.

Endoplasmic reticulum (ER) stress causes dysregulation of the cholesterol and triglyceride synthesis pathways by activating SREBP1 [69]. Arsenic induces ER stress in a variety of tissues and cell types [70]–[74]. Thus, induction of ER stress in liver by arsenic exposure would be expected. Our data suggest that the ER stress is induced in the livers long after the prenatal arsenic exposure has ended. This post-exposure response is consistent with an alteration in developmental program of the liver resulting in either a persistent induction of ER stress or priming for later induction by a second agent such as the hyperlipidemia that develops in these mice as they age. The common embryonic lineage of liver and pancreas suggests that the prenatal arsenic exposure may have similar effects in pancreatic cells. Thus, study of these events in pancreatic beta cells may provide important information regarding the linkage between arsenic exposure and diabetes mellitus.

In summary, our results clearly indicate that the prenatal arsenic exposure has dysregulated the developmental program of the liver as reflected in the gene expression profile differences. The gene expression changes with no obvious signs of liver inflammation suggest a pro-inflammatory state. We speculate that this pro-inflammatory state is the first hit in a two hit process leading to chronic low grade inflammation in the liver. The resulting liver disease likely provides the source of inflammation that triggers accelerated and exacerbated atherosclerosis in ApoE^−/−^ mice prenatally exposed to arsenic. Our published studies of livers of C57Bl/6 mice chronically exposed to arsenic also found little histological evidence of liver injury in unchallenged mice [75]. It was only after challenge with lipopolysaccharide (LPS) that we observed dramatic evidence of a hyper-response in the livers of arsenic-exposed mice. Thus, it is not surprising that there is no apparent histological difference in the livers of these unchallenged mice after transplacental arsenic exposure. The gene expression studies show linkages to chronic systemic inflammatory diseases, rheumatoid arthritis and diabetes mellitus. Both of these disease processes contribute to increased atherogenesis. We identified a potential new role for SREBP1 in regulating genes associated with these chronic systemic inflammatory diseases. SREBP1 is activated by ER stress suggesting that SREBP1 may be a common mediator of systemic inflammation for agents that induce ER stress. In order to understand the mechanism of altered development leading to accelerated atherogenesis, it will be important to compare gene expression profiles at multiple ages from fetal stage to adulthood and to confirm the role of SREBP1 in the initiation of systemic inflammation.

## Materials and Methods

### Ethics Statement

All procedures were performed in strict accordance with the recommendations in the Guide for the Care and Use of Laboratory Animals of the National Institutes of Health under protocols 03151 and 06093 approved by the University of Louisville Institutional Animal Care and Use Committee.

### Mice and Exposure Protocols

Apolipoprotein E-knockout mice (strain B6.129P2-Apoe^tm1Unc^/J) were purchased from Jackson Laboratories and maintained in an AALAAC approved vivarium as previously described [21]. Mice were bred by housing two females with a male. Females were checked daily for the presence of a vaginal plug which was designated gestational day 0 (GD0). Plug-positive females were caged separately until weaning at postnatal day (PND) 21. Dams were provided drinking water containing 85 mg/L sodium arsenite (49 ppm As) *ad libitum* from GD8 through birth. Arsenic containing water was changed twice weekly. After birth, dams were provided normal tap water *ad libitum*. Adult mice (10 weeks old, PND70) were euthanized by CO_2_ asphyxiation and newborns (PND1) by decapitation. Livers were quickly excised and snap frozen in liquid nitrogen.

### Measurement of Liver Arsenic Levels

Pregnant dams and fetuses were euthanized on GD18 by CO_2_ asphyxiation and decapitation, respectively. Livers were excised quickly and snap frozen. Three dams were included in each maternal group, and one male fetus from each of the pregnant dams was included in the fetal group. Small samples of frozen maternal and GD18 fetal livers (300–600 mg) were transferred to 2 mL acid washed (0.1 M HNO_3_ acid) centrifuge tubes and digested in 350 µL concentrated nitric acid overnight. One hundred µL of digested sample was transferred to 10 mL acid washed microwavable digestion tubes in triplicates, and every 3^rd^ sample from each group was spiked with 5 ng arsenic standards (SPEX CertiPrep, Metuchen, NJ). The samples were microwave digested at 150°C for 10 min using Automated Microwave Synthesis Workstation. Residues were removed by centrifugation, and 1.9 mL of 18 mega-ohm water containing 10 ppb internal standard solution was added in to every sample to give 5% HNO_3_ acid. Each sample (1.5 mL) was transferred to acid washed polypropylene deep-well 96 well plates for ICP-MS analysis using Thermo Electron ICP-MS, X-Series (in the Center for Regulatory, Environmental and Analytical Metabolomics at University of Louisville). Blank was concentrated nitric acid. Results are expressed in ng arsenic/g wet weight liver.

### RNA Preparation, Probe Preparations and Microarrays

Using mirVANA RNA purification kits (Ambion, Carlsbad, CA), total RNAs were purified from samples of frozen livers from three newborn (PND1) and three 10-week old mice (PND70) that had been exposed or unexposed to arsenic *in utero*. Each mouse was from a separate litter. RNA was quantitated and integrity determined on a Nanochip using a Bioanalyzer (Agilent, Santa Clara, CA). Microarray analyses for microRNA abundance in total liver RNA was performed by Exiqon (Woburn, MA) using Exiqon LNA 1500 microarrays in a two-color design against mouse standard total RNA (Ambion, now part of Applied Biosystems). Microarray analyses for mRNA abundance was performed using 2 color design against mouse standard total RNA (Stratgene, LaJolla, CA) on NIA Mouse 44K Microarray v2.1 (Whole Genome 60-mer Oligo: Agilent #2514117 [76]). Probe preparation, hybridization, scanning of arrays and log2 normalization of data were as described [76].

### Microarray Data Analyses

Microarray data were mean-centered and quantile normalized to normalize gene expression distribution across the samples. The ratiometric values (test/reference) were then transformed to log2 and lowess smoothing. The data were then subjected to two-way ANOVA (treatment, developmental time at p<0.01) for changes induced by arsenic exposure in PND1 and PND70 mice using Partek Genomics Suite v 6.3 (http://www.partek.com/). There were 763 significant probes corresponding to 712 genes with significant expression changes for PND70 samples and 848 probes corresponding to 797 genes for PND1 samples in the mRNA microarray dataset. The intersection of these two gene sets yielded 51 genes that were differentially expressed at both ages. Unsupervised hierarchical clustering and principal component analysis were performed using Partek software. Gene ontology annotation (Tables S1, S2, S3, S4) was performed using DAVID [77], [78] and interaction networks based on published literature were prepared using Partek. Enriched transcription factor binding sites were detected using the Expander 4.1 software [79], [80]. In order to detect enrichment, the background gene set was chosen from the total gene list downloaded from GEO [81] for NIA Mouse 44K Microarray V2.1 (Whole Genome 60-mer Oligo; accession GPL2552). Additional analyses of disease functions were performed using Ingenuity Pathway Analysis software. Microarray data have been deposited in the NCBI Gene Expression Omnibus Series record GSE30783 and are in compliance with MIAME guidelines.

### Identification of miRNA Targets

Gene targets of differentially expressed miRNAs were detected using *Mus musculus* miRBase v5, which lists the targets in terms of Ensembl transcripts. Ensembl gene transcripts and corresponding gene identifiers were downloaded from BioMart using Ensembl 52 and *Mus musculus* genes NCBIM37. All miRNA targets were then converted to Ensembl gene identifiers which could then be directly compared to the resulting differentially expressed mRNA.

### Detection of Enriched Transcription Factors

Enriched transcription factors were detected using the Expander 4.1 software [82], [83]. For each of the subsets, the Entrez gene identifiers were uploaded as a gene set. These gene sets were analyzed for transcription factor enrichment using the PRIMA software tool within Expander. In order to detect enrichment, the background gene set was chosen from the total gene list downloaded from GEO [84] for NIA Mouse 44K Microarray V2.1 (Whole Genome 60-mer Oligo; accession GPL2552).

A list of the transcription factors significantly enriched given the corresponding gene sets, as well as the number of genes within the set regulated by those transcription factors along with the P-Value (cutoff of 0.05) and enrichment factor are reported.

### Plasma Liver Enzyme Assay

Blood was drawn by cardiac puncture of anesthetized mice into collection tubes for preparation of plasma. Sodium citrate was used as an anticoagulant. Plasma levels of alanine aminotransferase (ALT) and aspartate aminotransferase (AST) were measured using commercially available kits (Infinity Liquid Reagents, Thermo Electron Corporation, Pittsburgh, PA).

### Western Blot Analysis of Liver Proteins

Liver samples obtained from mice derived from 7 different litters were homogenized in lysis buffer [10 mM Tris-HCl pH 7.4, 1 mM EDTA, 0.1% (w/v) SDS, 1 mM phenylmethylsulphonylfluoride (PMSF)] for protein isolation. Protein concentrations were determined by bicinchoninic acid (BCA) protein assay (Thermo Scientific, Rockford, IL.). Proteins were separated by SDS-PAGE (12% acrylamide), electroblotted to nitrocellulose, and probed with antibodies to HSC70 (rat mAb, 1∶1000, Assay Designs/Stressgen, Plymouth Meeting, PA.), HSP70i (mouse mAb, 1∶10,000, Assay Designs/Stressgen, Plymouth Meeting, PA), SREBP1 (rabbit mAb, 1∶1000; Abcam, Cambridge, MA), and their corresponding secondary antibodies (anti-rat, anti-mouse and anti-rabbit) conjugated to horseradish peroxidase (HRP). The signals were detected using chemiluminescence (ECL) substrate (Pierce, Rockford, IL) and exposure to Kodak XAR x-ray film. Quantification was performed with Image Quant software.

## Supporting Information

Figure S1 Interactome of mRNAs suppressed in livers of PND70 mice exposed to arsenic prenatal. Major hubs include HNF4A, IL8 and calcium signaling.
(TIF)

Figure S2 Interactome of mRNAs induced in livers of arsenic exposed PND70 mice. Major hubs include NFKB1, TNF, IL6 and IL8. Minor hubs include TLR4, LTB, LTA, CHUK, IL1A, HSPA1B and HSPA8.
(TIF)

Table S1 GO annotation analysis of the mRNAs with expression induced in PND1 livers of in utero arsenic exposed mice were analyzed by DAVID to identify which pathways were represented.
(DOCX)

Table S2 GO annotation analysis of the mRNAs with expression suppressed in PND1 livers of in utero arsenic exposed mice were analyzed by DAVID to identify which pathways were represented.
(DOCX)

Table S3 GO annotation analysis of the mRNAs with expression induced in PND70 livers of in utero arsenic exposed mice were analyzed by DAVID to identify which pathways were represented.
(DOCX)

Table S4 GO annotation analysis of the mRNAs with expression suppressed in PND70 livers of in utero arsenic exposed mice were analyzed by DAVID to identify which pathways were represented.
(DOCX)

Table S5 Gene promoters of differentially expressed mRNAs that are targets of microRNAs suppressed in arsenic exposed PND1 mice were analyzed for transcription factor binding sites. A total of 64 unique Entrez gene IDs are gene targets of down regulated microRNA and appear in the gene list of differentially expressed mRNAs at PND1. A total 15 transcription factors are enriched for this gene set with a P-value <0.05.
(DOCX)

Table S6 Gene promoters of differentially expressed mRNAs that are targets of microRNAs either induced or suppressed in arsenic exposed PND1 mice were analyzed for transcription factor binding sites. A total of 101 unique Entrez gene IDs are gene targets of up or down regulated microRNA and appear in the gene list of differentially expressed mRNAs at PND1. A total 17 transcription factors are enriched for this gene set with a P-value <0.05.
(DOCX)

Table S7 Gene promoters of differentially expressed mRNAs that are targets of microRNAs both induced and suppressed in arsenic exposed PND1 mice were analyzed for transcription factor binding sites. A total of 12 unique Entrez gene IDs are gene targets of both up AND down regulated microRNA and appear in the gene list of differentially expressed mRNAs at PND1. A total 11 transcription factors are enriched for this gene set with a P-value <0.05.
(DOCX)

Table S8 Gene promoters of differentially expressed mRNAs that are targets of microRNAs induced in arsenic exposed PND1 mice were analyzed for transcription factor binding sites. A total of 49 unique Entrez gene IDs are gene targets of up regulated microRNA and appear in the gene list of differentially expressed mRNAs at PND1. A total 10 transcription factors are enriched for this gene set with a P-value <0.05.
(DOCX)

Table S9 Gene promoters of differentially expressed mRNAs that are targets of microRNAs induced in arsenic exposed PND70 mice were analyzed for transcription factor binding sites. A total of 33 unique Entrez gene IDs are gene targets of up regulated microRNA and appear in the gene list of differentially expressed mRNAs at PND70. A total 13 transcription factors are enriched for this gene set with a P-value <0.05.
(DOCX)

Table S10 Gene promoters of differentially expressed mRNAs that are targets of microRNAs suppressed in arsenic exposed PND70 mice were analyzed for transcription factor binding sites. A total of 124 unique entrez gene IDs are gene targets of down regulated miRNA and appear in the gene list of differentially expressed mRNAs at PND70. A total 21 transcription factors are enriched for this gene set.with a P-value <0.05.
(DOCX)

Table S11 Gene promoters of differentially expressed mRNAs that are targets of microRNAs either induced or suppressed in arsenic exposed PND70 mice were analyzed for transcription factor binding sites. A total of 135 unique entrez gene IDs are gene targets of up OR down regulated miRNA and appear in the gene list of differentially expressed mRNAs at PND70. A total 28 transcription factors are enriched for this gene set.with a P-value <0.05.
(DOCX)

Table S12 Gene promoters of differentially expressed mRNAs that are targets of microRNAs both induced and suppressed in arsenic exposed PND70 mice were analyzed for transcription factor binding sites. A total of 22 unique entrez gene IDs are gene targets of BOTH up AND down regulated miRNA and appear in the gene list of differentially expressed mRNAs at PND70. A total 9 transcription factors are enriched for this gene set.with a P-value <0.05.
(DOCX)
