# Supplementary figures and images for: Prenatal Arsenic Exposure Alters Gene Expression in the Adult Liver to a Proinflammatory State Contributing to Accelerated Atherosclerosis
Source: PLoS One. 2012 Jun 15;7(6):e38713. doi: 10.1371/journal.pone.0038713 (PMC3376138; doi:10.1371/journal.pone.0038713)

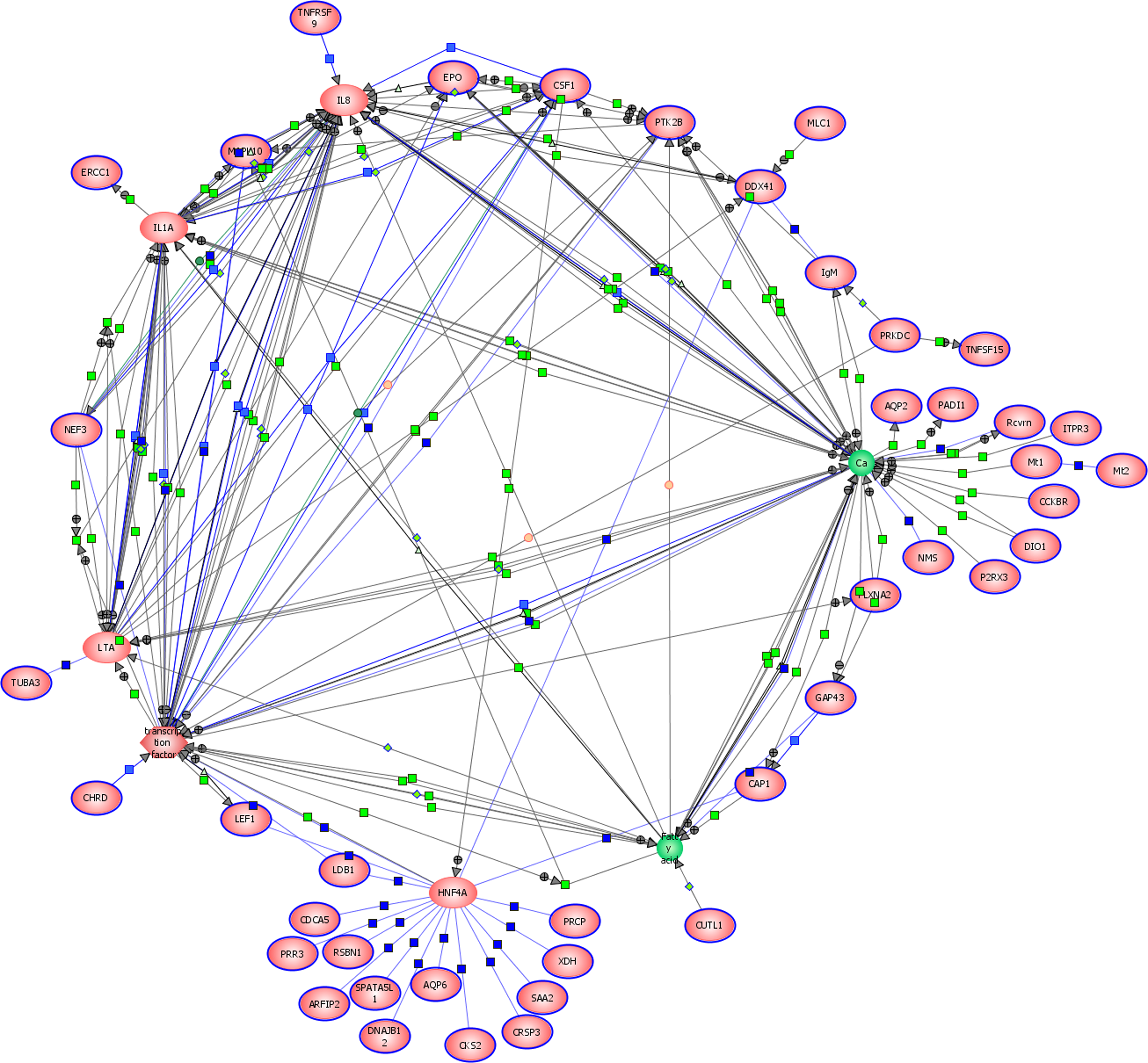

Supplement: Figure S1 — Interactome of mRNAs suppressed in livers of PND70 mice exposed to arsenic prenatal. Major hubs include HNF4A, IL8 and calcium signaling. (TIF) [file pone.0038713.s001.tif]

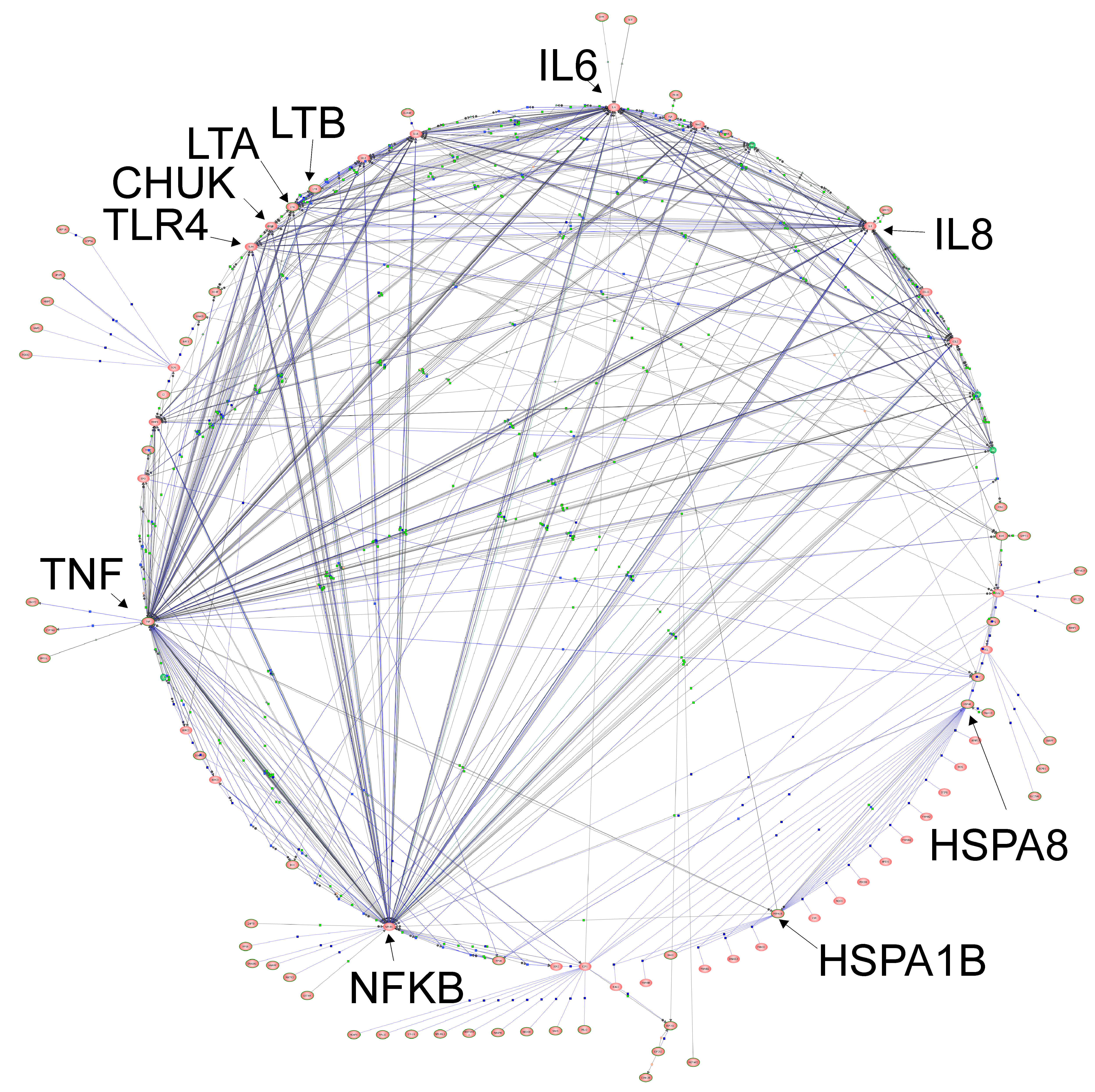

Supplement: Figure S2 — Interactome of mRNAs induced in livers of arsenic exposed PND70 mice. Major hubs include NFKB1, TNF, IL6 and IL8. Minor hubs include TLR4, LTB, LTA, CHUK, IL1A, HSPA1B and HSPA8. (TIF) [file pone.0038713.s002.tif]
