# Supplementary material for: Prenatal Arsenic Exposure Alters Gene Expression in the Adult Liver to a Proinflammatory State Contributing to Accelerated Atherosclerosis
Source: PLoS One. 2012 Jun 15;7(6):e38713. doi: 10.1371/journal.pone.0038713 (PMC3376138; doi:10.1371/journal.pone.0038713)
Supplement: Table S1 — GO annotation analysis of the mRNAs with expression induced in PND1 livers of in utero arsenic exposed mice were analyzed by DAVID to identify which pathways were represented. (DOCX) [file pone.0038713.s003.docx]

**Table S1. Gene Ontology of mRNAs induced by arsenic exposure in PND1 mice**

| **Category** | **Term** | **Count** | **%** | **PValue** |
| --- | --- | --- | --- | --- |
| GOTERM_BP_4 | nucleobase, nucleoside, nucleotide and nucleic acid metabolism | 67 | 18.6% | 1.95E-04 |
| GOTERM_BP_4 | biopolymer metabolism | 54 | 15.0% | 0.001 |
| GOTERM_MF_4 | nuclease activity | 9 | 2.5% | 0.002 |
| GOTERM_BP_4 | cellular biosynthesis | 27 | 7.5% | 0.003 |
| GOTERM_BP_4 | embryonic development (sensu Vertebrata) | 6 | 1.7% | 0.006 |
| GOTERM_MF_4 | transition metal ion binding | 44 | 12.2% | 0.009 |
| GOTERM_BP_4 | vitamin metabolism | 5 | 1.4% | 0.011 |
| GOTERM_BP_4 | protein metabolism | 56 | 15.6% | 0.018 |
| GOTERM_CC_4 | intracellular membrane-bound organelle | 98 | 27.2% | 0.023 |
| GOTERM_BP_4 | macromolecule biosynthesis | 17 | 4.7% | 0.025 |
| GOTERM_BP_4 | cellular macromolecule metabolism | 53 | 14.7% | 0.026 |
| GOTERM_CC_4 | nucleus | 68 | 18.9% | 0.027 |
| GOTERM_BP_4 | regulation of cellular metabolism | 43 | 11.9% | 0.037 |
| GOTERM_BP_4 | cellular catabolism | 12 | 3.3% | 0.056 |
| GOTERM_BP_4 | RNA-mediated posttranscriptional gene silencing | 2 | 0.6% | 0.070 |
| GOTERM_BP_4 | transport | 49 | 13.6% | 0.072 |
| GOTERM_BP_4 | macromolecule catabolism | 9 | 2.5% | 0.082 |
| GOTERM_MF_4 | transferase activity, transferring groups other than amino-acyl groups | 6 | 1.7% | 0.082 |
