# Supplementary material for: Prenatal Arsenic Exposure Alters Gene Expression in the Adult Liver to a Proinflammatory State Contributing to Accelerated Atherosclerosis
Source: PLoS One. 2012 Jun 15;7(6):e38713. doi: 10.1371/journal.pone.0038713 (PMC3376138; doi:10.1371/journal.pone.0038713)
Supplement: Table S2 — GO annotation analysis of the mRNAs with expression suppressed in PND1 livers of in utero arsenic exposed mice were analyzed by DAVID to identify which pathways were represented. (DOCX) [file pone.0038713.s004.docx]

**Table S2. Gene Ontology of mRNAs suppressed by arsenic exposure in PND1 mice**

| **Category** | **Term** | **Count** | **%** | **PValue** |
| --- | --- | --- | --- | --- |
| GOTERM_BP_4 | generation of precursor metabolites and energy | 20 | 6.1% | 0.001 |
| GOTERM_BP_4 | carbohydrate metabolism | 13 | 3.9% | 0.011 |
| GOTERM_BP_4 | lipid metabolism | 14 | 4.2% | 0.038 |
| GOTERM_BP_4 | alkene metabolism | 3 | 0.9% | 0.044 |
| GOTERM_BP_4 | alcohol metabolism | 8 | 2.4% | 0.045 |
| GOTERM_CC_4 | endoplasmic reticulum | 16 | 4.9% | 0.008 |
| GOTERM_CC_4 | endosome | 5 | 1.5% | 0.034 |
| GOTERM_MF_4 | calcium ion binding | 22 | 6.7% | 0.017 |
| GOTERM_MF_4 | phosphotransferase activity, nitrogenous group as acceptor | 3 | 0.9% | 0.093 |
| KEGG_PATHWAY | MMU00010:GLYCOLYSIS / GLUCONEOGENESIS | 6 | 1.8% | 0.004 |
| KEGG_PATHWAY | HSA04910:INSULIN SIGNALING PATHWAY | 2 | 0.6% | 0.073 |
| KEGG_PATHWAY | HSA04720:LONG-TERM POTENTIATION | 2 | 0.6% | 0.073 |
| KEGG_PATHWAY | MMU05040:HUNTINGTON'S DISEASE | 3 | 0.9% | 0.085 |
| KEGG_PATHWAY | MMU00460:CYANOAMINO ACID METABOLISM | 2 | 0.6% | 0.091 |
