# Supplementary material for: Prenatal Arsenic Exposure Alters Gene Expression in the Adult Liver to a Proinflammatory State Contributing to Accelerated Atherosclerosis
Source: PLoS One. 2012 Jun 15;7(6):e38713. doi: 10.1371/journal.pone.0038713 (PMC3376138; doi:10.1371/journal.pone.0038713)
Supplement: Table S3 — GO annotation analysis of the mRNAs with expression induced in PND70 livers of in utero arsenic exposed mice were analyzed by DAVID to identify which pathways were represented. (DOCX) [file pone.0038713.s005.docx]

**Table S3. Gene Ontology of mRNAs induced by arsenic exposure in PND70 mice**

| **Category** | **Term** | **Count** | **%** | **PValue** |
| --- | --- | --- | --- | --- |
| GOTERM_CC_4 | intracellular membrane-bound organelle | 111 | 33.4% | 2.01E-06 |
| GOTERM_MF_4 | translation initiation factor activity | 7 | 2.1% | 3.85E-04 |
| KEGG_PATHWAY | MMU03060:PROTEIN EXPORT | 4 | 1.2% | 4.32E-04 |
| GOTERM_CC_4 | endoplasmic reticulum | 19 | 5.7% | 5.17E-04 |
| GOTERM_BP_4 | establishment of cellular localization | 21 | 6.3% | 0.002 |
| GOTERM_BP_4 | cellular localization | 21 | 6.3% | 0.002 |
| GOTERM_CC_4 | membrane coat | 5 | 1.5% | 0.004 |
| GOTERM_CC_4 | vesicular fraction | 8 | 2.4% | 0.004 |
| GOTERM_BP_4 | vesicle-mediated transport | 13 | 3.9% | 0.005 |
| GOTERM_BP_4 | biopolymer metabolism | 52 | 15.7% | 0.006 |
| GOTERM_CC_4 | Golgi apparatus | 15 | 4.5% | 0.006 |
| GOTERM_BP_4 | response to heat | 4 | 1.2% | 0.009 |
| GOTERM_CC_4 | intracellular non-membrane-bound organelle | 31 | 9.3% | 0.011 |
| KEGG_PATHWAY | MMU03010:RIBOSOME | 6 | 1.8% | 0.012 |
| GOTERM_CC_4 | chromatin | 7 | 2.1% | 0.015 |
| GOTERM_CC_4 | ribosome | 8 | 2.4% | 0.021 |
| GOTERM_CC_4 | vesicle membrane | 5 | 1.5% | 0.022 |
| GOTERM_CC_4 | cytoplasmic membrane-bound vesicle | 8 | 2.4% | 0.022 |
| GOTERM_CC_4 | cytoplasmic vesicle | 8 | 2.4% | 0.029 |
| GOTERM_CC_4 | signal recognition particle (sensu Eukaryota) | 3 | 0.9% | 0.035 |
| GOTERM_CC_4 | signal recognition particle | 3 | 0.9% | 0.035 |
| GOTERM_BP_4 | transport | 52 | 15.7% | 0.038 |
| GOTERM_CC_4 | extrinsic to plasma membrane | 4 | 1.2% | 0.043 |
| KEGG_PATHWAY | MMU04612:ANTIGEN PROCESSING AND PRESENTATION | 5 | 1.5% | 0.044 |
| KEGG_PATHWAY | HSA03010:RIBOSOME | 3 | 0.9% | 0.046 |
| GOTERM_CC_4 | chromosome | 9 | 2.7% | 0.050 |
| GOTERM_CC_4 | sex chromosome | 2 | 0.6% | 0.055 |
| KEGG_PATHWAY | MMU03022:BASAL TRANSCRIPTION FACTORS | 3 | 0.9% | 0.065 |
| GOTERM_CC_4 | nuclear chromosome | 4 | 1.2% | 0.069 |
| GOTERM_CC_4 | perinuclear region | 3 | 0.9% | 0.071 |
| GOTERM_CC_4 | nucleus | 63 | 19.0% | 0.072 |
| GOTERM_BP_4 | DNA repair | 6 | 1.8% | 0.096 |
