# Supplementary material for: Prenatal Arsenic Exposure Alters Gene Expression in the Adult Liver to a Proinflammatory State Contributing to Accelerated Atherosclerosis
Source: PLoS One. 2012 Jun 15;7(6):e38713. doi: 10.1371/journal.pone.0038713 (PMC3376138; doi:10.1371/journal.pone.0038713)
Supplement: Table S4 — GO annotation analysis of the mRNAs with expression suppressed in PND70 livers of in utero arsenic exposed mice were analyzed by DAVID to identify which pathways were represented. (DOCX) [file pone.0038713.s006.docx]

**Table S4. Gene Ontology of mRNAs suppressed by arsenic exposure in PND70 mice**

| **Category** | **Term** | **Count** | **%** | **PValue** |
| --- | --- | --- | --- | --- |
| GOTERM_BP_4 | DNA repair | 7 | 2.7% | 0.003 |
| GOTERM_BP_4 | homophilic cell adhesion | 5 | 1.9% | 0.009 |
| GOTERM_MF_4 | calcium ion binding | 16 | 6.1% | 0.010 |
| GOTERM_CC_4 | intracellular non-membrane-bound organelle | 20 | 7.7% | 0.012 |
| GOTERM_BP_4 | odontogenesis | 3 | 1.2% | 0.019 |
| GOTERM_BP_4 | M phase | 6 | 2.3% | 0.020 |
| GOTERM_BP_4 | mitotic cell cycle | 6 | 2.3% | 0.022 |
| GOTERM_BP_4 | ossification | 4 | 1.5% | 0.026 |
| GOTERM_BP_4 | bone remodeling | 4 | 1.5% | 0.033 |
| GOTERM_CC_4 | cytoskeleton | 12 | 4.6% | 0.060 |
| GOTERM_CC_4 | intermediate filament cytoskeleton | 4 | 1.5% | 0.063 |
| GOTERM_MF_4 | cytokine activity | 6 | 2.3% | 0.074 |
| GOTERM_CC_4 | chromosome | 6 | 2.3% | 0.078 |
| GOTERM_BP_4 | intracellular signaling cascade | 13 | 5.0% | 0.097 |
| GOTERM_MF_4 | GTPase binding | 3 | 1.2% | 0.097 |
| GOTERM_BP_4 | fluid transport | 2 | 0.8% | 0.098 |
| GOTERM_MF_4 | guanyl-nucleotide exchange factor activity | 4 | 1.5% | 0.098 |
