# Supplementary material for: Prenatal Arsenic Exposure Alters Gene Expression in the Adult Liver to a Proinflammatory State Contributing to Accelerated Atherosclerosis
Source: PLoS One. 2012 Jun 15;7(6):e38713. doi: 10.1371/journal.pone.0038713 (PMC3376138; doi:10.1371/journal.pone.0038713)
Supplement: Table S5 — Gene promoters of differentially expressed mRNAs that are targets of microRNAs suppressed in arsenic exposed PND1 mice were analyzed for transcription factor binding sites. A total of 64 unique Entrez gene IDs are gene targets of down regulated microRNA and appear in the gene list of differentially expressed mRNAs at PND1. A total 15 transcription factors are enriched for this gene set with a P-value <0.05. (DOCX) [file pone.0038713.s007.docx]

**Table S5: Transcription factor binding sites enriched in gene promoters of differentially expressed mRNAs that are targets of microRNAs suppressed in arsenic exposed PND1 mice**

| **Transcription Factor** | **Number of Genes** | **P-Value** | **Enrichment Factor** |
| --- | --- | --- | --- |
| **M00649[MAZ]** | 20 | 0.0090 | 1.876 |
| **M00731[Osf2]** | 11 | 0.0010 | 2.978 |
| **M00205[GR]** | 8 | 0.035 | 1.936 |
| **M01109[SZF1-1]** | 11 | 0.011 | 1.908 |
| **M00490[Bach2]** | 10 | 0.046 | 1.977 |
| **M00339[c-Ets-1]** | 12 | 0.046 | 1.565 |
| **M00395[HOXA3]** | 6 | 0.0060 | 2.271 |
| **M00467[Roaz]** | 10 | 0.0030 | 2.265 |
| **M01033[HNF4]** | 34 | 0.015 | 1.299 |
| **M00678[Tel-2]** | 9 | 0.012 | 1.738 |
| **M01119[KAISO]** | 8 | 0.036 | 1.92 |
| **M00721[CACCC-binding_factor]** | 15 | 0.03 | 1.788 |
| **M00025[Elk-1]** | 14 | 0.031 | 1.582 |
| **M00441[GBF]** | 9 | 0.033 | 1.691 |
| **M00088[Ik-3]** | 9 | 0.023 | 2.338 |
