# Supplementary material for: Prenatal Arsenic Exposure Alters Gene Expression in the Adult Liver to a Proinflammatory State Contributing to Accelerated Atherosclerosis
Source: PLoS One. 2012 Jun 15;7(6):e38713. doi: 10.1371/journal.pone.0038713 (PMC3376138; doi:10.1371/journal.pone.0038713)
Supplement: Table S6 — Gene promoters of differentially expressed mRNAs that are targets of microRNAs either induced or suppressed in arsenic exposed PND1 mice were analyzed for transcription factor binding sites. A total of 101 unique Entrez gene IDs are gene targets of up or down regulated microRNA and appear in the gene list of differentially expressed mRNAs at PND1. A total 17 transcription factors are enriched for this gene set with a P-value <0.05. (DOCX) [file pone.0038713.s008.docx]

**Table S6: Transcription factor binding sites enriched in gene promoters of differentially expressed mRNAs that are targets of microRNAs either induced or suppressed in arsenic exposed PND1 mice**

| **Transcription Factor** | **Number of Genes** | **P-Value** | **Enrichment Factor** |
| --- | --- | --- | --- |
| **M01045[AP-2alphaA]** | 18 | 0.042 | 1.731 |
| **M00649[MAZ]** | 28 | 0.003 | 1.72 |
| **M00731[Osf2]** | 13 | 0.006 | 2.304 |
| **M00976[AHRHIF]** | 27 | 0.009 | 1.556 |
| **M00205[GR]** | 11 | 0.040 | 1.743 |
| **M01109[SZF1-1]** | 14 | 0.031 | 1.59 |
| **M00490[Bach2]** | 17 | 0.004 | 2.2 |
| **M00395[HOXA3]** | 8 | 0.009 | 1.982 |
| **M00467[Roaz]** | 12 | 0.007 | 1.779 |
| **M00076[GATA-2]** | 13 | 0.021 | 2.012 |
| **M01033[HNF4]** | 47 | 0.022 | 1.176 |
| **M00797[HIF-1]** | 28 | 0.031 | 1.547 |
| **M00678[Tel-2]** | 12 | 0.027 | 1.517 |
| **M00172[AP-1]** | 15 | 0.009 | 1.826 |
| **M00652[Nrf-1]** | 28 | 0.036 | 1.377 |
| **M00484[Ncx]** | 12 | 0.042 | 1.859 |
| **M00378[Pax-4]** | 21 | 0.046 | 1.343 |
