# Supplementary material for: Prenatal Arsenic Exposure Alters Gene Expression in the Adult Liver to a Proinflammatory State Contributing to Accelerated Atherosclerosis
Source: PLoS One. 2012 Jun 15;7(6):e38713. doi: 10.1371/journal.pone.0038713 (PMC3376138; doi:10.1371/journal.pone.0038713)
Supplement: Table S7 — Gene promoters of differentially expressed mRNAs that are targets of microRNAs both induced and suppressed in arsenic exposed PND1 mice were analyzed for transcription factor binding sites. A total of 12 unique Entrez gene IDs are gene targets of both up AND down regulated microRNA and appear in the gene list of differentially expressed mRNAs at PND1. A total 11 transcription factors are enriched for this gene set with a P-value <0.05. (DOCX) [file pone.0038713.s009.docx]

**Table S7: Transcription factor binding sites enriched in gene promoters of differentially expressed mRNAs that are targets of microRNAs both induced and suppressed in arsenic exposed PND1 mice**

| **Transcription Factor** | **Number of Genes** | **P-Value** | **Enrichment Factor** |
| --- | --- | --- | --- |
| **M00731[Osf2]** | 3 | 0.041 | 4.06 |
| **M00271[AML-1a]** | 9 | 0.028 | 1.878 |
| **M01109[SZF1-1]** | 3 | 0.038 | 2.602 |
| **M00395[HOXA3]** | 2 | 0.017 | 3.785 |
| **M00394[Msx-1]** | 3 | 0.039 | 4.167 |
| **M00678[Tel-2]** | 4 | 0.028 | 3.863 |
| **M00172[AP-1]** | 5 | 0.001 | 4.648 |
| **M00052[NF-kappaB_(p65)]** | 3 | 0.030 | 4.88 |
| **M00453[IRF-7]** | 4 | 0.036 | 3.534 |
| **M00086[Ik-1]** | 3 | 0.007 | 4.167 |
| **M00322[c-Myc:Max]** | 5 | 0.035 | 2.201 |
