# Supplementary material for: Prenatal Arsenic Exposure Alters Gene Expression in the Adult Liver to a Proinflammatory State Contributing to Accelerated Atherosclerosis
Source: PLoS One. 2012 Jun 15;7(6):e38713. doi: 10.1371/journal.pone.0038713 (PMC3376138; doi:10.1371/journal.pone.0038713)
Supplement: Table S8 — Gene promoters of differentially expressed mRNAs that are targets of microRNAs induced in arsenic exposed PND1 mice were analyzed for transcription factor binding sites. A total of 49 unique Entrez gene IDs are gene targets of up regulated microRNA and appear in the gene list of differentially expressed mRNAs at PND1. A total 10 transcription factors are enriched for this gene set with a P-value <0.05. (DOCX) [file pone.0038713.s010.docx]

**Table S8: Transcription factor binding sites enriched in gene promoters of differentially expressed mRNAs that are targets of microRNAs induced in arsenic exposed PND1 mice**

| **Transcription Factor** | **Number of Genes** | **P-Value** | **Enrichment Factor** |
| --- | --- | --- | --- |
| **M00976[AHRHIF]** | 14 | 0.028 | 1.694 |
| **M00641[HSF]** | 8 | 0.012 | 2.009 |
| **M00490[Bach2]** | 10 | 0.0070 | 2.718 |
| **M00706[TFII-I]** | 11 | 0.017 | 1.745 |
| **M00395[HOXA3]** | 4 | 0.049 | 2.081 |
| **M00514[ATF4]** | 9 | 0.037 | 1.695 |
| **M00172[AP-1]** | 11 | 4.98E-4 | 2.812 |
| **M00652[Nrf-1]** | 14 | 0.034 | 1.446 |
| **M00484[Ncx]** | 8 | 0.017 | 2.602 |
| **M00453[IRF-7]** | 9 | 0.015 | 2.186 |
