# Supplementary material for: Prenatal Arsenic Exposure Alters Gene Expression in the Adult Liver to a Proinflammatory State Contributing to Accelerated Atherosclerosis
Source: PLoS One. 2012 Jun 15;7(6):e38713. doi: 10.1371/journal.pone.0038713 (PMC3376138; doi:10.1371/journal.pone.0038713)
Supplement: Table S9 — Gene promoters of differentially expressed mRNAs that are targets of microRNAs induced in arsenic exposed PND70 mice were analyzed for transcription factor binding sites. A total of 33 unique Entrez gene IDs are gene targets of up regulated microRNA and appear in the gene list of differentially expressed mRNAs at PND70. A total 13 transcription factors are enriched for this gene set with a P-value <0.05. (DOCX) [file pone.0038713.s011.docx]

**Table S9: Transcription factor binding sites enriched in gene promoters of differentially expressed mRNAs that are targets of microRNAs induced in arsenic exposed PND70 mice**

| **Transcription Factor** | **Number of Genes** | **P-Value** | **Enrichment Factor** |
| --- | --- | --- | --- |
| **M01045[AP-2alphaA]** | 12 | 6.69E-4 | 3.029 |
| **M00801[CREB]** | 7 | 0.014 | 1.797 |
| **M00492[STAT1]** | 7 | 0.036 | 2.482 |
| **M00805[LEF1]** | 20 | 0.024 | 1.323 |
| **M00641[HSF]** | 8 | 0.023 | 2.512 |
| **M00056[myogenin_/_NF-1]** | 6 | 0.033 | 2.071 |
| **M00626[RFX1_(EF-C)]** | 9 | 0.0090 | 2.726 |
| **M00940[E2F-1]** | 7 | 0.029 | 2.23 |
| **M00161[Oct-1]** | 7 | 0.035 | 2.148 |
| **M00346[GATA-1]** | 6 | 0.013 | 3.383 |
| **M00033[p300]** | 6 | 0.042 | 2.011 |
| **M00017[ATF]** | 11 | 0.014 | 2.061 |
| **M00803[E2F]** | 25 | 7.26E-4 | 2.151 |
