# Supplementary material for: Prenatal Arsenic Exposure Alters Gene Expression in the Adult Liver to a Proinflammatory State Contributing to Accelerated Atherosclerosis
Source: PLoS One. 2012 Jun 15;7(6):e38713. doi: 10.1371/journal.pone.0038713 (PMC3376138; doi:10.1371/journal.pone.0038713)
Supplement: Table S10 — Gene promoters of differentially expressed mRNAs that are targets of microRNAs suppressed in arsenic exposed PND70 mice were analyzed for transcription factor binding sites. A total of 124 unique entrez gene IDs are gene targets of down regulated miRNA and appear in the gene list of differentially expressed mRNAs at PND70. A total 21 transcription factors are enriched for this gene set.with a P-value <0.05. (DOCX) [file pone.0038713.s012.docx]

**Table S10: Transcription factor binding sites enriched in gene promoters of differentially expressed mRNAs that are targets of microRNAs suppressed in arsenic exposed PND70 mice**

| **Transcription Factor** | **# of Genes** | **P-Value** | **Enrichment Factor** |
| --- | --- | --- | --- |
| **M00403[aMEF-2]** | 17 | 0.041 | 1.741 |
| **M00055[N-Myc]** | 26 | 0.019 | 1.53 |
| **M00801[CREB]** | 18 | 0.047 | 1.344 |
| **M00695[ETF]** | 40 | 0.012 | 1.43 |
| **M00189[AP-2]** | 44 | 0.038 | 1.357 |
| **M00056[myogenin_/_NF-1]** | 15 | 0.019 | 1.506 |
| **M00626[RFX1_(EF-C)]** | 19 | 0.037 | 1.674 |
| **M00062[IRF-1]** | 18 | 0.038 | 1.574 |
| **M00940[E2F-1]** | 19 | 0.0060 | 1.761 |
| **M00938[E2F-1]** | 40 | 2.99E-4 | 1.497 |
| **M00341[GABP]** | 27 | 0.034 | 1.277 |
| **M00326[Pax-1]** | 11 | 0.04 | 1.679 |
| **M00652[Nrf-1]** | 38 | 0.015 | 1.428 |
| **M00430[E2F-1]** | 13 | 0.0030 | 1.447 |
| **M00128[GATA-1]** | 15 | 0.0010 | 2.279 |
| **M00033[p300]** | 19 | 0.0040 | 1.853 |
| **M00025[Elk-1]** | 23 | 0.015 | 1.3 |
| **M00223[STATx]** | 14 | 0.04 | 1.646 |
| **M00803[E2F]** | 58 | 0.0040 | 1.452 |
| **M00441[GBF]** | 15 | 0.045 | 1.409 |
| **M00287[NF-Y]** | 31 | 5.55E-4 | 1.511 |
