# Supplementary material for: Prenatal Arsenic Exposure Alters Gene Expression in the Adult Liver to a Proinflammatory State Contributing to Accelerated Atherosclerosis
Source: PLoS One. 2012 Jun 15;7(6):e38713. doi: 10.1371/journal.pone.0038713 (PMC3376138; doi:10.1371/journal.pone.0038713)
Supplement: Table S11 — Gene promoters of differentially expressed mRNAs that are targets of microRNAs either induced or suppressed in arsenic exposed PND70 mice were analyzed for transcription factor binding sites. A total of 135 unique entrez gene IDs are gene targets of up OR down regulated miRNA and appear in the gene list of differentially expressed mRNAs at PND70. A total 28 transcription factors are enriched for this gene set.with a P-value <0.05. (DOCX) [file pone.0038713.s013.docx]

**Table S11: Transcription factor binding sites enriched in gene promoters of differentially expressed mRNAs that are targets of microRNAs either induced or suppressed in arsenic exposed PND70 mice**

| **Transcription Factor** | **Number of Genes** | **P-Value** | **Enrichment Factor** |
| --- | --- | --- | --- |
| **M00055[N-Myc]** | 29 | 0.013 | 1.565 |
| **M01045[AP-2alphaA]** | 23 | 0.0090 | 1.548 |
| **M00801[CREB]** | 21 | 0.015 | 1.438 |
| **M00492[STAT1]** | 19 | 0.023 | 1.796 |
| **M00446[Spz1]** | 23 | 0.034 | 1.587 |
| **M00976[AHRHIF]** | 31 | 0.045 | 1.25 |
| **M00915[AP-2]** | 44 | 0.027 | 1.318 |
| **M00695[ETF]** | 44 | 0.0050 | 1.442 |
| **M00189[AP-2]** | 51 | 0.022 | 1.442 |
| **M00224[STAT1]** | 16 | 0.044 | 1.716 |
| **M00056[myogenin_/_NF-1]** | 16 | 0.022 | 1.472 |
| **M00626[RFX1_(EF-C)]** | 22 | 0.015 | 1.776 |
| **M00062[IRF-1]** | 20 | 0.027 | 1.603 |
| **M00940[E2F-1]** | 22 | 0.0020 | 1.869 |
| **M00938[E2F-1]** | 43 | 3.31E-4 | 1.475 |
| **M00341[GABP]** | 29 | 0.043 | 1.257 |
| **M00139[AhR]** | 26 | 0.039 | 1.448 |
| **M00326[Pax-1]** | 12 | 0.035 | 1.679 |
| **M00778[AhR]** | 30 | 0.015 | 1.677 |
| **M00652[Nrf-1]** | 41 | 0.011 | 1.412 |
| **M00430[E2F-1]** | 14 | 0.0030 | 1.428 |
| **M00800[AP-2]** | 44 | 0.049 | 1.304 |
| **M00128[GATA-1]** | 16 | 0.0010 | 2.228 |
| **M00033[p300]** | 20 | 0.0020 | 1.788 |
| **M00025[Elk-1]** | 24 | 0.026 | 1.243 |
| **M00803[E2F]** | 66 | 0.0010 | 1.514 |
| **M00441[GBF]** | 17 | 0.031 | 1.464 |
| **M00287[NF-Y]** | 33 | 6.2E-4 | 1.474 |
