# Supplementary material for: Prenatal Arsenic Exposure Alters Gene Expression in the Adult Liver to a Proinflammatory State Contributing to Accelerated Atherosclerosis
Source: PLoS One. 2012 Jun 15;7(6):e38713. doi: 10.1371/journal.pone.0038713 (PMC3376138; doi:10.1371/journal.pone.0038713)
Supplement: Table S12 — Gene promoters of differentially expressed mRNAs that are targets of microRNAs both induced and suppressed in arsenic exposed PND70 mice were analyzed for transcription factor binding sites. A total of 22 unique entrez gene IDs are gene targets of BOTH up AND down regulated miRNA and appear in the gene list of differentially expressed mRNAs at PND70. A total 9 transcription factors are enriched for this gene set.with a P-value <0.05. (DOCX) [file pone.0038713.s014.docx]

**Table S12: Transcription factor binding sites enriched in gene promoters of differentially expressed mRNAs that are targets of microRNAs both induced and suppressed in arsenic exposed PND70 mice**

| **Transcription Factor** | **Number of Genes** | **P-Value** | **Enrichment Factor** |
| --- | --- | --- | --- |
| **M01045[AP-2alphaA]** | 6 | 0.033 | 2.203 |
| **M00733[SMAD-4]** | 5 | 0.035 | 2.268 |
| **M00805[LEF1]** | 13 | 0.023 | 1.251 |
| **M00641[HSF]** | 6 | 0.032 | 2.74 |
| **M00056[myogenin_/_NF-1]** | 5 | 0.018 | 2.51 |
| **M00626[RFX1_(EF-C)]** | 6 | 0.036 | 2.643 |
| **M00346[GATA-1]** | 4 | 0.043 | 3.28 |
| **M00017[ATF]** | 7 | 0.035 | 1.908 |
| **M00803[E2F]** | 17 | 0.0020 | 2.128 |
